# Heterologous Expression of a Cryptic Gene Cluster from Streptomyces leeuwenhoekii C34^T^ Yields a Novel Lasso Peptide, Leepeptin

**DOI:** 10.1128/AEM.01752-19

**Published:** 2019-11-14

**Authors:** Juan Pablo Gomez-Escribano, Jean Franco Castro, Valeria Razmilic, Scott A. Jarmusch, Gerhard Saalbach, Rainer Ebel, Marcel Jaspars, Barbara Andrews, Juan A. Asenjo, Mervyn J. Bibb

**Affiliations:** a Department of Molecular Microbiology, John Innes Centre, Norwich, United Kingdom; b Centre for Biotechnology and Bioengineering (CeBiB), Department of Chemical Engineering and Biotechnology, Universidad de Chile, Santiago, Chile; c Marine Biodiscovery Centre, Department of Chemistry, University of Aberdeen, Old Aberdeen, United Kingdom; University of Manchester

**Keywords:** *Streptomyces*, natural products, RiPP, lasso peptide, heterologous expression

## Abstract

Analysis of the genome sequence of Streptomyces leeuwenhoekii C34^T^ identified biosynthetic gene clusters (BGCs) for three different lasso peptides (Lp1, Lp2, and Lp3) which were not known to be made by the strain. Lasso peptides represent relatively new members of the RiPP (ribosomally synthesized and posttranslationally modified peptides) family of natural products and have not been extensively studied. Lp3, whose production could be detected in culture supernatants from S. leeuwenhoekii C34^T^ and after heterologous expression of its BGC in Streptomyces coelicolor, is identical to the previously characterized chaxapeptin. Lp1, whose production could not be detected or achieved heterologously, appears to be identical to a recently identified member of the citrulassin family of lasso peptides. Since production of Lp2 by S. leeuwenhoekii C34^T^ was not observed, its BGC was also expressed in S. coelicolor. The lasso peptide was isolated and its structure confirmed by mass spectrometry and nuclear magnetic resonance analyses, revealing a novel structure that appears to represent a new family of lasso peptides.

**IMPORTANCE** Recent developments in genome sequencing combined with bioinformatic analysis have revealed that actinomycetes contain a plethora of unexpected BGCs and thus have the potential to produce many more natural products than previously thought. This reflects the inability to detect the production of these compounds under laboratory conditions, perhaps through the use of inappropriate growth media or the absence of the environmental cues required to elicit expression of the corresponding BGCs. One approach to overcoming this problem is to circumvent the regulatory mechanisms that control expression of the BGC in its natural host by deploying heterologous expression. The generally compact nature of lasso peptide BGCs makes them particularly amenable to this approach, and, in the example given here, analysis revealed a new member of the lasso peptide family of RiPPs. This approach should be readily applicable to other cryptic lasso peptide gene clusters and would also facilitate the design and production of nonnatural variants by changing the sequence encoding the core peptide, as has been achieved with other classes of RiPPs.

## Abstract (precis)

Recent developments in genome sequencing combined with bioinformatic analysis have revealed that actinomycetes contain a plethora of unexpected BGCs and thus have the potential to produce many more natural products than previously thought. This reflects the inability to detect the production of these compounds under laboratory conditions, perhaps through the use of inappropriate growth media or the absence of the environmental cues required to elicit expression of the corresponding BGCs. One approach to overcoming this problem is to circumvent the regulatory mechanisms that control expression of the BGC in its natural host by deploying heterologous expression. The generally compact nature of lasso peptide BGCs makes them particularly amenable to this approach, and, in the example given here, analysis revealed a new member of the lasso peptide family of RiPPs. This approach should be readily applicable to other cryptic lasso peptide gene clusters and would also facilitate the design and production of nonnatural variants by changing the sequence encoding the core peptide, as has been achieved with other classes of RiPPs.

## INTRODUCTION

A group of strains belonging to the bacterial genus *Streptomyces*, subsequently named Streptomyces leeuwenhoekii, were isolated from a sample collected from the Chaxa Lagoon in the Salar de Atacama, an extreme environment located in northern Chile (1, 2). These strains have been shown to produce a range of previously unknown natural products. For example, S. leeuwenhoekii C38 produces the polyketide atacamycin (3); S. leeuwenhoekii C58 produces the lasso peptide chaxapeptin (4); S. leeuwenhoekii C79 produces four new specialized metabolites (H.-P. Fiedler, unpublished results); and the type species, S. leeuwenhoekii C34^T^ (2), produces two groups of polyketides: the chaxamycins (5) and the chaxalactins (6). The chaxamycins and the chaxalactins both possess antibacterial activity, while chaxamycin also displayed potential anticancer activity by inhibiting the chaperone protein Hsp90; the atacamycins show inhibitory activity toward phosphodiesterase PDE-4B2 (and thus may have anti-inflammatory potential) and, in the case of atacamycin A, antiproliferative activity against adeno carcinoma and breast carcinoma cells; chaxapeptin showed inhibitory activity toward human lung cancer cell line A549. Thus, despite few studies, it appears that streptomycetes (and probably other *Actinobacteria*) isolated from this extreme and little-scrutinized ecological niche are likely to prove to be an abundant source of new natural products with a range of potentially useful biological activities.

Our previous analysis of the genome sequence of S. leeuwenhoekii C34^T^ revealed the presence of 35 biosynthetic gene clusters (BGCs), including 3 encoding the putative lasso peptides Lp1, Lp2, and Lp3 (7, 8). Lasso peptides have been isolated from a wide range of bacteria, including *Burkholderia*, *Caulobacter*, *Enterobacteriaceae*, *Rhodococcus*, and *Streptomyces* species derived from terrestrial, freshwater, and marine environments (see reference 9 and references therein), although recent bioinformatic analysis suggests a much broader phylogenetic distribution (10). They represent a growing class of bioactive bacterial peptides, forming a subfamily within the much larger RiPP (ribosomally synthesized and posttranslationally modified peptides) family of natural products (11), and are characterized by a specific knotted structure, the lasso fold (12). Lasso peptides are produced initially as precursor peptides with an N-terminal leader sequence and a C-terminal retained core region of 14 to 24 amino acid residues (13) that contains a macrocyclic ring formed between the carboxylate side chain of an Asp or Glu residue located at position 7, 8, or 9 of the core peptide and the amino group of the N-terminal amino acid residue. While it was initially thought that there was a requirement for Gly to be the first core peptide residue, examples of lasso peptides with N-terminal Ala, Ser, and Cys have also been reported, and a recent bioinformatic analysis revealed even greater flexibility at this position (10). The C-terminal tail is threaded through the macrocycle, presumably prior to ring closure, and is generally retained in place sterically by the presence of C-terminal residues with bulky side chains (although disulfide bonds play that role in some lasso peptides). The resulting highly compact structure confers thermal stability and resistance to proteases and results in a broad range of biological activities that include enzyme inhibition and receptor antagonism; some lasso peptides possess antibiotic activity (13). The unusual lasso structure, which has thus far proved inaccessible to peptide synthesis, has also attracted attention as a scaffold for epitope grafting (14), providing the opportunity to develop novel lasso peptide-derived biological activities with therapeutic potential.

Lasso peptide biosynthesis requires a lasso cyclase (C-protein), homologous to asparagine synthase, and a leader peptidase (B-protein), homologous to transglutaminase. Biosynthesis also requires a RiPP recognition element (RRE [an E-protein]; note that we have adopted the nomenclature used in reference 10 for this protein, rather than the previously used B1) which binds the leader peptide and directs enzymatic modification. In most cases (an exception is provided in this work), lasso peptide BGCs also encode transporters for product export (15).

Here, we describe the analysis of three lasso peptide gene clusters of S. leeuwenhoekii C34^T^ and the heterologous expression of two of them, leading to the chemical characterization of one of the molecules, leepeptin, as a novel lasso peptide.

## RESULTS

### Identification of three lasso peptide gene clusters in the S. leeuwenhoekii C34^T^ genome sequence.

**FIG 1:**
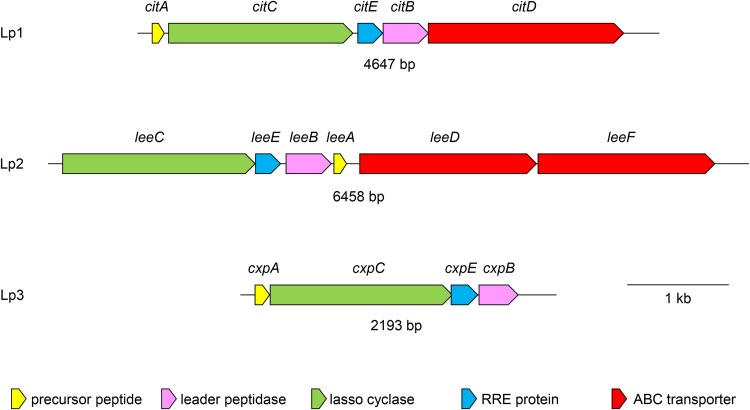
Schematic representation of the three lasso peptide gene clusters of S. leeuwenhoekii C34^T^. Since the product of the Lp1 gene cluster appears to be identical to one of the members of the citrulassin family of lasso peptides, the genes have been named *cit*; similarly, since the product of the Lp3 gene cluster was shown to be chaxapeptin, the genes have been named *cxp*.

Three putative lasso peptide BGCs were identified in the genome sequence of S. leeuwenhoekii C34^T^ (7). These BGCs were not identified at the time by antiSMASH (16) but were revealed by searching initially for homologues of the lasso cyclase, LarB, involved in lariatin biosynthesis in Rhodococcus jostii (GenBank accession number BAL72547.1) (17) using blastP and the deduced proteome of S. leeuwenhoekii C34^T^. Manual inspection of the genome sequences adjacent to identified homologues revealed, in three cases, all of the enzymatic functions required for the biosynthesis of three different lasso peptides. Moreover, the lack of substantial intergenic regions suggested that all of the biosynthetic genes for each peptide might be contained in a single transcription unit. A schematic representation of the BGCs is shown in Fig. 1. While the BGCs for Lp1 and Lp2 are chromosomally located (GenBank accession number LN831790), that for Lp3 occurs on pSLE2 (GenBank accession number LN831789) and thus is potentially capable of lateral transfer by conjugation.

### Identification of Lp3 as chaxapeptin.

Supernatants of S. leeuwenhoekii C34^T^ cultures grown in five different media were analyzed by matrix-assisted laser desorption ionization–time of flight (MALDI-ToF) mass spectrometry (MS) for the production of the expected peptides. When this work was initiated in early 2014, with the exception of one example of the use of Cys (10), Gly was the only residue known to be found at the N terminus of lasso core peptides; this apparent constraint influenced our predictions of the size of the mature peptides made by each of the three BGCs. The Lp1 BGC encodes the precursor peptide MKKAYEAPTLVRLGSFRRKT*GLLQRSGNDRLILSKN* (the core residues that we predicted, indicated in italics, would yield a molecule with an accurate mass of 1,764.9959 Da after cyclization). The Lp2 BGC encodes the precursor peptide MEHDEKTPYETPAVYGLGAFAEET*GLYGVRNDEEINWHFDYWT* (the initially predicted core sequence, in italics, would yield a molecule with an accurate mass of 2,395.0658 Da after cyclization). The Lp3 BGC encodes the precursor peptide MTELQPEAYEAPSLIEVGEFSEDTL*GFGSKPLDSFGLNFF* (the predicted core residues, in italics, would yield a molecule with an accurate mass of 1,613.7879 Da after cyclization). Ions with masses matching those expected for Lp3 were readily detected and in high abundance (see Fig. S1 in the supplemental material); we could not detect any ions corresponding to the expected peptides for Lp1 and Lp2 or ions that could correspond to slightly different peptides (e.g., one residue more or less if we had not predicted the core peptide correctly).

Since Lp3 was readily detected in most of the culture media tested, we focused our initial attention on this peptide. Sequencing of the peptide using liquid chromatography–tandem high-resolution mass spectrometry (LC/tandem-HRMS) on a Synapt G2Si platform (Waters) revealed the expected sequence of the C-terminal tail and identified the only aspartate in the core peptide as the residue likely to be involved in the macrocyclization (Fig. S2), as we had predicted (we could not detect fragmentation of the macrocycle, and so the last a, b, and y ions detected indicated the residue likely involved in ring formation with the N-terminal NH_2_). These results, shown in more detail in the supplemental material, led us to conclude that Lp3 is identical to chaxapeptin produced by the closely related strain S. leeuwenhoekii C58 (4). Lp3 is also similar to sungsanpin (11/15 identical core residues), which is produced by a marine actinomycete, *Streptomyces* sp. strain SNJ013, that had been isolated from deep sea sediment collected off the Korean coast (9). A blastP search of the GenBank database also revealed homologues of Lp3 in the genome sequences of both Streptomyces kanamyceticus (GenBank accession number WP_107099006) and Streptomyces cinnamoneus (accession number WP_104531718) (Fig. S3).

### Heterologous expression of the BGCs in S. coelicolor.

The BGC for Lp3 is unusual in that it does not contain any gene encoding a possible transporter; it was unclear to us whether Lp3 could be secreted by ABC transporters encoded by the other two lasso peptide BGCs or by a nonspecific transporter encoded somewhere else in the genome. In contrast, the Lp2 BGC has two genes for possible ABC transporters, while that for Lp1 encodes a single ABC transporter. The three BGCs were cloned in the expression vector pIJ10257 (18) downstream from the constitutive ermE* promoter (19) and were transferred to Streptomyces coelicolor expression hosts M1152 and M1154 (20) by conjugation (see the supplemental material for details).

We could readily detect Lp3 in culture supernatants of the heterologous hosts containing the Lp3 expression construct pIJ12815 by both MALDI-ToF and liquid chromatography–ion trap–time of flight mass spectrometry (LC-IT-ToF MS) (Fig. S4); the molecule had the same accurate mass and peptide sequence (determined by LC/tandem-HRMS on a Waters Synapt G2Si platform) as Lp3 produced by S. leeuwenhoekii C34^T^. Since S. coelicolor does not contain any lasso peptide BCGs, this result suggests that export of Lp3 is not dependent on a dedicated lasso peptide transporter. We also transferred pIJ12815 to Streptomyces albus J1074 and found that we could also readily detect production of mature Lp3 in the culture supernatant (Fig. S4). These results suggest that the secretion of mature Lp3 can be mediated by a nonspecific transporter generally present in *Streptomyces* species.

**FIG 2:**
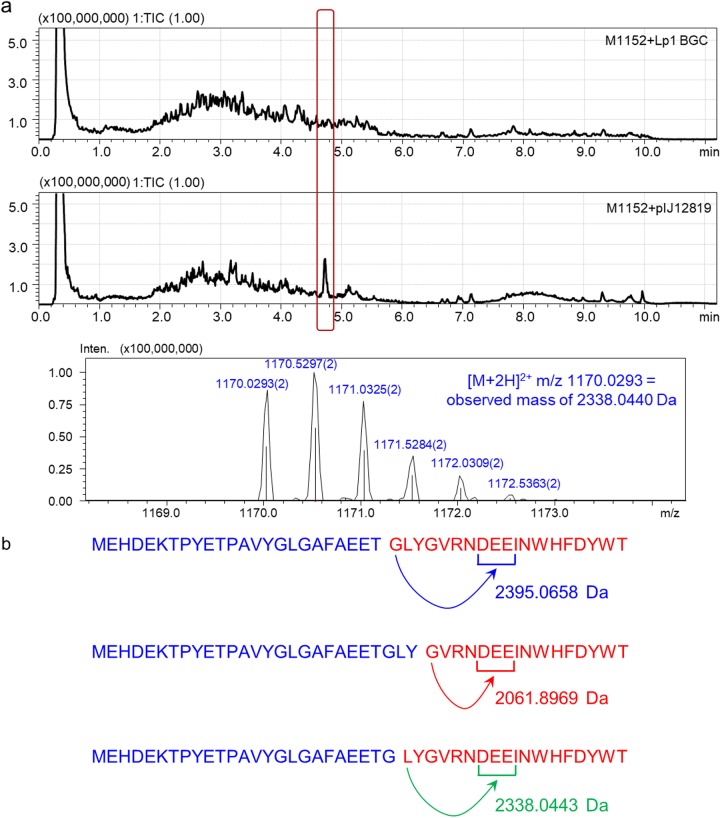

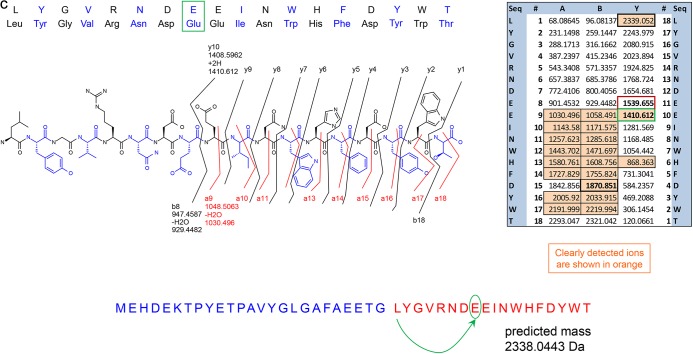
(a) Heterologous expression of the Lp2 BGC (contained in pIJ12819) in S. coelicolor M1152. Total ion chromatogram (TIC) and zoomed-in full spectrum of the highlighted peak obtained by LC-IT-ToF MS (Shimadzu). The detected ion, [M + 2H]^2+^, with 1,170.0293 *m*/*z* suggests an observed neutral mass of 2,338.0440 Da for the monoisotopic molecule. (b) Possible core peptides deduced from the nucleotide sequence of the *leeA* precursor peptide gene. Core peptides are shown in red and leader sequences in blue; the arrows indicate possible cyclization events. (c) Ions expected and observed after LC/tandem-HRMS analysis of leepeptin. The table of expected ions was calculated for the linear peptide after subtraction of a water molecule from the ions represented in panels b and a (using http://db.systemsbiology.net:8080/proteomicsToolkit/FragIonServlet.html). The ions that were clearly detected are shaded in orange. The predicted structure of leepeptin is shown in red at the bottom, with the green arrow indicating cyclization between Leu_1_ and Glu_8_ (the leader sequence is shown in blue). Seq, sequence.

The Lp2 BGC is also unusual in that the precursor peptide gene (*leeA*) lies downstream of the genes encoding biosynthetic activities (*leeCEB*) (Fig. 1). Our first expression construct contained just *leeCEBA* transcribed from the constitutive ermE* promoter; transfer to heterologous hosts S. coelicolor M1152 and M1154 failed to yield the expected molecule or one of similar mass (data not shown). We then added the two putative ABC transporter genes *leeDF*, reconstructing the native gene arrangement (*leeCEBADF*; see Materials and Methods and Fig. S5 for details). Transfer of the resulting pIJ12819 plasmid to S. coelicolor M1152 and M1154 resulted in the production of a molecule (2,338.0440 Da) that was 57 Da lower in mass than that we had predicted for Lp2 (2,395.0658 Da) (Fig. 2a). Structure elucidation of this molecule (see below) resolved this anomaly and confirmed that the observed molecule was the product of the Lp2 BGC (Fig. S11). These results indicated that, in contrast to Lp3 (chaxapeptin), the two ABC transporters encoded within the BGC are required for peptide secretion (we did not test whether one of the genes would suffice).

Attempts to detect production of Lp1 by both S. leeuwenhoekii C34^T^ (MALDI-ToF analyses only; see Fig. S1) and S. coelicolor after introduction of the BCG (MALDI-ToF and LC-IT-ToF MS analyses; data not shown) failed. However, multiple blastP searches of the GenBank database performed using the predicted amino sequences of the Lp1 biosynthetic enzymes and ABC transporter as query sequences identified several *Streptomyces* strains that could potentially produce a similar lasso peptide. Four of these strains (Streptomyces viridochromogenes DSM 40736, Streptomyces davaonensis JCM 4913, Streptomyces pristinaspiralis ATCC 25486, and Streptomyces roseochromogenes subsp. *oscitans* DS 12.976) were grown in a variety of different media, but we could not detect any possible candidate peptides in culture supernatants. Since these attempts were made, it has become apparent that the predicted core peptide of Lp1 is identical to that of one of the members of the citrulassin family of lasso peptides identified in reference 10. Interestingly, the study reported in reference 10 demonstrated that for family member citrulassin A, Arg9 of the core sequence (LLGLAGNDRLVLSKN) is modified to citrulline. Arg9 is conserved across the large citrulassin family of lasso peptides (10), suggesting that this unusual modification may occur in many if not all family members. As those published data established Lp1 as a member of a previously identified class of lasso peptide, we abandoned further attempts to express its BGC heterologously.

### Characterization of Lp2 by liquid chromatography—high-resolution mass spectrometry (LC-HRMS) and LC/tandem-HRMS.

Heterologous expression of the Lp2 BGC in S. coelicolor produced a molecule with a mass of 2,338.0440 Da (the monoisotopic ion detected by LC-IT-ToF MS was 1,170.0293 *m*/*z* for [M + 2H]^2+^) (Fig. 2a). This molecule was present in culture supernatants from all S. coelicolor M1152 and M1154 derivatives containing the Lp2 BGC but absent in samples from the host without the BGC (in Fig. 2a, the negative control is M1152 carrying the Lp1 BGC). The Lp2 precursor peptide (MEHDEKTPYETPAVYGLGAFAEET*GLYGVRNDEEINWHFDYWT*) could yield several possible core peptides (Fig. 2b). At the time when we started this project in early 2014, with the single exception of Cys (10), Gly was the only amino acid known to be used to form the macrocyclic ring; consequently, we predicted two possible core peptides, namely, GLYGVRNDEEINWHFDYWT, which would yield a molecule with an accurate mass of 2,395.0658 Da after cyclization, and GVRNDEEINWHFDYWT, which would yield a molecule with an accurate mass of 2,061.8970 Da after cyclization. However, analysis of the molecule detected by LC-IT-ToF MS indicated that the core peptide was likely to be LYGVRNDEEINWHFDYWT. This matched, within the accepted range of instrument error, the predicted mass (2,338.0443 Da after cyclization) to the observed mass (2,338.0440 Da). The partial sequence of the peptide was observed by LC/tandem-HRMS on a Synapt G2Si platform (Waters); all the expected a, b, and y ions for the 10 C-terminal residues were clearly identified in the deconvoluted fragmentation spectrum (Fig. 2c; see also Fig. S6 and S7) confirming that the heterologously produced peptide was the product of the Lp2 BGC. We named this new natural product, with (at the time) an unprecedented N-terminal Leu, leepeptin.

The LC/tandem-HRMS fragmentation data also allowed us to identify the internal acidic residue likely to be involved in macrocyclization. Three acidic residues could potentially be involved based on the rules of lasso peptide macrocycle formation: Asp_7_, Glu_8_, and Glu_9_ (positions refer to the core peptide). The last clearly detected fragment ions were a^9^ (predicted, 1,030.4960; observed, 1,030.4958) and b^9^ (predicted, 1,058.4910; observed, 1,058.4921). We did not observe any ions that matched those expected for the a^8^, b^8^, and y^11^ ions. Altogether, these results suggested that it is the side chain of Glu_8_ that closes the macrocycle with the NH_2_ of the N-terminal Leu (Fig. 2c; see also Fig. S7).

### NMR structure elucidation of leepeptin.

All of the ^1^H and ^13^C nuclear magnetic resonance (NMR) data were in agreement with *in silico* and MS predictions. Along with one-dimensional (1D) experiments, 2D ^1^H-^13^C heteronuclear single quantum correlation (HSQC), ^1^H-^13^C heteronuclear multiple-bond correlation (HMBC), ^1^H-^1^H total correlated spectroscopy (TOCSY), ^1^H-^13^C HSQC-TOCSY, and ^1^H-^1^H nuclear Overhauser effect spectroscopy (NOESY) allowed assignment of nearly all hydrogens (see Table S2 in the supplemental material) in the 18-residue peptide, including two Trp, two Tyr, two Asn, two Asp., two Glu, one Leu, one Gly, one Val, one Arg, one Ile, one His, one Phe and one Thr.

**FIG 3:**
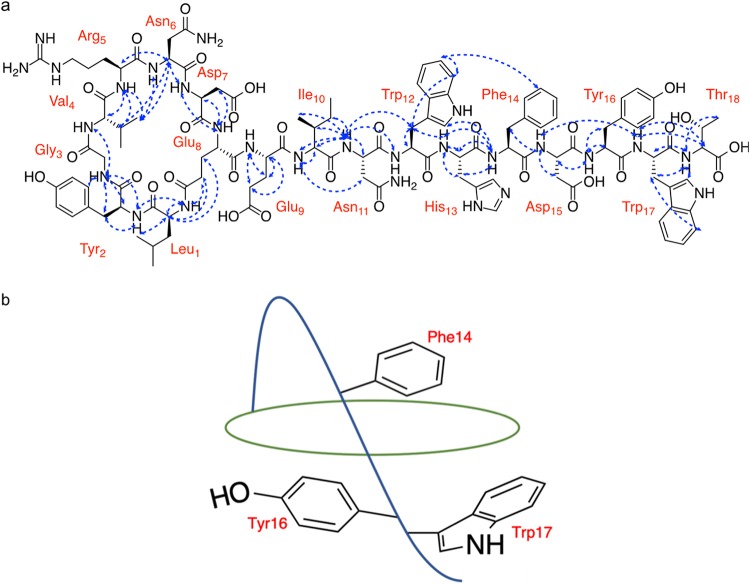
(a) NMR structure determination of leepeptin. Key NOESY correlations (blue) establishing sequence connectivity between amino acid residues. (b) Stylized representation of leepeptin. Tail residues deduced to be plugs, based on tail-to-ring NOE correlations, are displayed.

Sequence connectivity was established using NOESY correlations (Fig. 3a). The connection between Leu_1_ and Tyr_2_ was established using the correlations between Leu_1_-NH and Tyr_2_-NH. The connection between Tyr_2_ and Gly_3_ was established using the correlations among Tyr_2_-NH, αH, βH (δ 2.35), and δH (δ 6.71) and Gly_3_-NH. Furthermore, the Gly_3_-Val_4_ connection was determined using Gly_3_-NH to Val_4_-NH and Val_4_-βH correlations as well as the Gly_3_-αH (all) to Val_4_-NH correlations. The Val_4_-Arg_5_ connection was determined using Val_4_-NH, αH, βH, and γH (δ 0.95) correlations (all) to Arg_5_-NH correlations. The Arg_5_-Asn_6_ connection was established using Arg_5_-αH and Asn_6_-αH correlations as well as Val_4_-βH and γH (δ 0.95) connections to both Asn_6_-NH and Asn_6_-αH. Furthermore, the Asn_6_-Asp_7_ connection was determined using the Asn_6_-αH and Asp_7_-NH correlations. The Asp_7_-Glu_8_ connection was established using the Asp_7_-NH, αH, and βH (δ 2.27) correlations (all) to Glu_8_-NH and Asp_7_-NH to Glu_8_-βH (δ 1.75). The Glu_8_-Leu_1_ macrocyclization was determined through analysis of NOEs from Glu_8_-βH (δ 1.54) and γH (δ 1.67) to Leu_1_-αH as well as Glu_8_-γH (δ 1.67) to Leu_1_-NH.

There is no NMR evidence for the sequence connectivities of Glu_8_-Glu_9_ and Glu_9_-Ile_10_, but there is tandem MS (MS^2^) (Fig. 2c) evidence for these linkages. The Ile_10_-Asn_11_ connection was determined using the correlations between Ile_10_-NH to Asn_11_-βH (δ 2.66) and Ile_10_-NH, αH, βH, γH’s, and ε (δ 0.82) (all to Asn_11_-NH). Furthermore, the Asn_11_-Trp_12_ connection was established using the correlations between Asn_11_-NH to Trp_12_-NH and βH (δ 2.95). The Trp_12_-His_13_ connection was established using the correlations from Trp_12_-βH (δ 2.95) and His_13_-NH. The His_13_-Phe_14_ connection was determined using the correlations between His_13_-NH, αH, and βH (δ 1.05) to Phe_14_-NH. Furthermore, the Phe_14_-Asp_15_ connection was determined using the correlations between Phe_14_-βH’s to Asp_15_-NH. The Asp_15_-Tyr_16_ connection was determined using the correlation between Asp_15_-αH and Tyr_16_-NH. The Tyr_16_-Trp_17_ connection was difficult to assign using NOEs due to overlapping chemical shifts of multiple residues with those signals assigned in Trp_17_. The Tyr_16_-ε (δ 6.38) correlation to Trp_17_-NH and the longer-range correlation between Asp_15_-NH to Trp_17_-NH help to place this residue. Lastly, the Trp_17_-Thr_18_ connection was established using the correlations between Trp-αH, βH’s, and δH (δ 7.37) (all to Thr_18_-NH) as well as from Trp_17_-NH to Thr_18_-αH.

NOESY data are generally used to confirm the residues in class II lasso peptides that prevent unthreading of the structure (see reference 15 for a discussion of the different classes of lasso peptides). Purely on the basis of the number of NOE correlations from tail residues back to macrocyclic residues, we were able to predict which amino acid residues in the tail of the peptide play a role in locking the tail in place. Working from the C-terminal Thr, Trp17 and Tyr16 each contain seven NOE correlations (Fig. S8). Both of these two residues seem to act as lower plugs, in a “harpoon”-like fashion, which is unusual among class II lasso peptides (Fig. 3b). Further up the tail, Phe14 contains seven NOE correlations, suggesting that it acts as the upper plug (Fig. S8).

In an attempt to establish the absolute conﬁguration of the amino acid residues of leepeptin, the methodology used in determining the absolute stereochemistry of chaxapeptin was followed (4). Marfey’s analysis was used to determine the L conformation for all amino acids, with the exception of Leu_1_, Trp_12_ and Trp_17_ (overlapping peaks).

### Leepeptin represents a new subfamily of lasso peptides.

**FIG 4:**
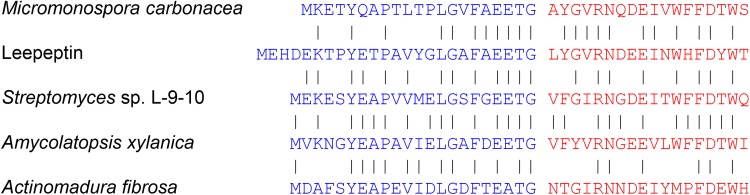
Alignment of the amino acid sequences of the precursor peptide of leepeptin (GenBank accession number CQR60041.1) with homologues found in the GenBank database, including Micromonospora carbonacea (SCF49478.1), *Streptomyces* sp. L-9-10 (RYJ28011.1), Amycolatopsis xylanica (SDZ36359.1), and Actinomadura fibrosa (WP_131760281.1). Leader sequences are shown in blue and core peptide sequences in red.

While we could not detect any close homologues of leepeptin in the data set reported in reference 10, blastP searches (21) of the GenBank database revealed four homologues encoded by the genome sequences of *Streptomyces* sp. L-9-10, Micromonospora carbonacea, Amycolatopsis xylanica, and Actinomadura fibrosa (Fig. 4). Generation of a sequence similarity network (SSN) (22) using the precursor peptide sequences identified in Data Set 2 in reference 10 but also including these four newly identified sequences confirmed the emergence of this new subfamily of lasso peptides (Fig. S9). blastP searches of precursor or core peptide data sets derived from reference 10 but also including these four newly identified sequences and using the precursor and core sequences of each of the five peptides as query sequences confirmed the results of the SSN analyses; the only significant hits were other members of this new subfamily (data not shown).

## DISCUSSION

Analysis of the genome sequence of S. leeuwenhoekii C34^T^ had revealed three BGCs with the potential to produce the lasso peptides Lp1, Lp2, and Lp3 (7). Each of these putative lasso peptides was also later identified at the sequence level in the bioinformatic analysis reported in reference 10 but had not been characterized further. Lp3 was shown in this work to be identical to the previously published chaxapeptin from the closely related strain S. leeuwenhoekii C58, also isolated from the Chaxa Lagoon in the Salar de Atacama. Production by S. leeuwenhoekii C34^T^ and, after heterologous expression, by S. coelicolor was detected. Unusually, no transporters were encoded by the Lp3 BGC, and the results of our heterologous expression experiments suggest that export of chaxapeptin is mediated by a nonspecific secretion system commonly present in *Streptomyces* species; we are not aware of any other experimental reports of a lack of a specific transporter for lasso peptide export. Genome sequence information is available for three of the four strains that possess genes encoding Lp3 homologues (see Fig. S3 in the supplemental material); while neither of the BGCs identified in S. leeuwenhoekii C58 and in S. kanamyceticus encodes identifiable transporters, that present in S. cinnamoneus encodes a two-component ABC transporter (data not shown). Why some members of this newly identified family of lasso peptides should apparently utilize a specific transporter while others do not is unknown.

Although we could not detect production of Lp1 by S. leeuwenhoekii C34^T^ or elicit its synthesis in S. coelicolor, the predicted product appears to be identical to a member of the recently identified citrulassin family of lasso peptides (10).

**FIG 5:**
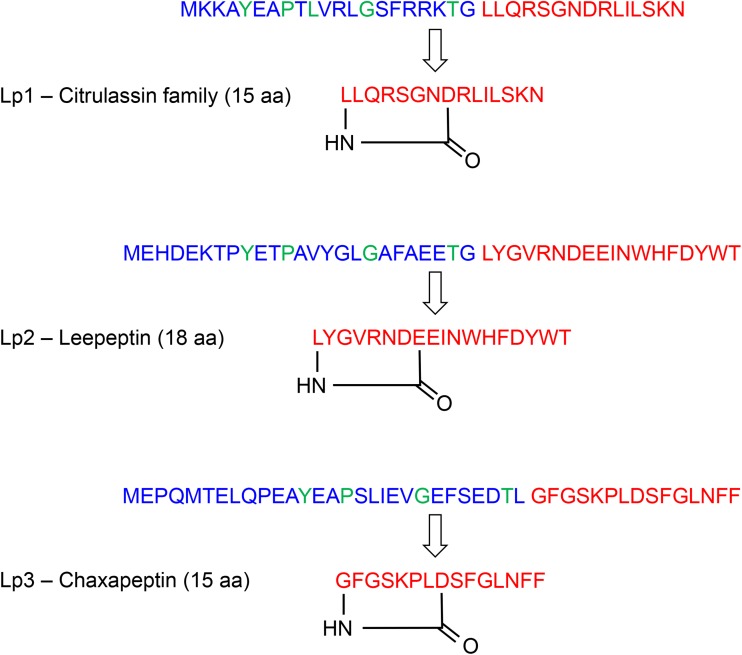
The three lasso peptides of S. leeuwenhoekii C34^T^. Leader sequences are shown in blue, with conserved leader residues in green; core peptide sequences before and after proteolytic cleavage and cyclization are shown in red.

Lp2, which appeared to represent a novel lasso peptide which we subsequently named leepeptin, could not be detected in the culture supernatant of S. leeuwenhoekii C34^T^ but could be readily isolated after heterologous expression in S. coelicolor, enabling its structural elucidation. Lack of production in the native strain but successful expression of the Lp2 BCG in the heterologous host under the control of the constitutive ermE* promoter presumably reflects lack of activation of the regulatory mechanism required for leepeptin biosynthesis in S. leeuwenhoekii C34^T^ under the growth conditions used. This may indicate that a specific environmental signal that is required to elicit leepeptin production in the native host was not provided under the experimental conditions used. Leepeptin appears to represent a new subfamily of lasso peptides. All three of the S. leeuwenhoekii C34^T^ lasso peptides contain the conserved leader sequence motif YxxPxLxxxGxxxxxTx, with the sole exception of the replacement of Leu_6_ with Val in the leader peptide of leepeptin, and all three leader peptides possess the “invariant” (with one known exception; see reference 10) Thr as the penultimate amino acid residue (Fig. 5).

Lasso peptides are currently an understudied family of natural products. Although the number of isolated compounds is relatively small (44 were identified in the supplementary information of reference 10), genome mining has revealed gene clusters for over 1,300 unique lasso peptides occurring across a wide range of bacterial genera (10). Over 35% of these BGCs are found in *Actinobacteria*, and many may not be expressed under laboratory conditions at the levels required for their detection. Consequently, we believe that the approach of heterologous expression described here could provide a useful means to unlock this untapped source of novel chemical diversity. Moreover, our discovery of a structurally novel lasso peptide, leepeptin (whose biological activity remains to be explored), in samples from the extreme environment of the Chaxa Lagoon in the high-altitude Atacama Desert provides further support for the hypothesis that the isolation of microorganisms from relatively little-explored environments such as this may result in a plethora of new natural products.

## MATERIALS AND METHODS

### Strains, culture conditions, and general methods.

Bacterial strains used and generated during this study are listed in Table 1. Escherichia coli and *Streptomyces* strains were cultured, maintained, and manipulated genetically following the methods described in reference 23 and reference 24, respectively. Spore stocks of S. leeuwenhoekii were prepared following standard methods from cultures grown on mannitol soya flour (SFM) agar medium at 37°C for 5 to 7 days. The culture media used for lasso peptide production were as follows: R3 (25), TY (TSB [tryptone soya broth; Oxoid CM0129]–YEME [yeast extract-malt extract; 24] [50:50]), LPM (Lariatin production medium) (17), LS (lassomycin seed medium) (26), and modified ISP2 (5). Plasmids and oligonucleotides used or constructed during this work are listed in Table 2 and Table S1 in the supplemental material, respectively. Molecular biology enzymes, reagents, and kits were used according to the manufacturers’ instructions. High-fidelity PCR amplification was performed with Phusion or Q5 DNA polymerases following the instructions of the manufacturer (NEB, Ipswich, MA) with nucleotide proportions of 15A/15T/35G/35C to improve the amplification efficiency of high-moles-percent (mol%) G+C *Streptomyces* DNA. Sanger sequencing was performed using the Eurofins Genomics service (Ebersberg, Germany) and analyzed using the Staden package, version 2.0.0b9 (http://staden.sourceforge.net) (27). blastP searches were performed at the NCBI server. Drawing of chemical structures and mass calculations were performed with Accelrys Draw 4.1 SP1 (Accelrys Inc.) and ChemDraw Professional 18 (PerkinElmer, Inc.). NMR data were analyzed with MestreNova V12 (Mestrelab Research S.L.).

**TABLE 1:** Bacterial strains used in this study

Strain	Description	Reference(s) and/or source(s)
E. coli DH5α	Strain used for routine cloning	29
E. coli ET12567/pUZ8002	Methylation-deficient strain used for conjugation with *Streptomyces*; pUZ8002 provides conjugation machinery	E. coli ET12567, 30; pUZ8002, David Figurski, personal communication
S. leeuwenhoekii C34^T^	Type strain	2
S. coelicolor M1152	M145, Δ*act* Δ*red* Δ*cpk* Δ*cda rpoB*[C1298T]	20
S. coelicolor M1154	M145, Δ*act* Δ*red* Δ*cpk* Δ*cda rpoB*[C1298T] *rpsL*[A262G]	20
S. albus J1074	S. albus G derivative J1074 (*ilv-1 sal-2* R^−^ M^−^)	31
S. viridochromogenes DSM 40736	Wild-type strain	NCBI reference sequence; NZ_GG657757.1
S. pristinaspiralis ATCC 25486	Wild-type strain	NCBI reference sequence; NZ_CM000950.1
S. davawensis JCM 4913	Wild-type strain	32 (strain also known as S. davaonensis JCM 4913^T^)
S. roseochromogenes subsp. *oscitans* DS 12.976	Wild-type strain	33
S. coelicolor M1623	S. coelicolor M1152/pIJ12819	This work

**TABLE 2:** Plasmids used and constructed during this study

Plasmid	Description	Reference or source
pBluescript II KS(+)	General cloning vector	34
pIJ10257	Expression vector for *Streptomyces*, with ermE*p, *hyg*, conjugative (*oriT* from RK2), integrative (phiC31 *attP*)	18
pIJ12815	pIJ10257 with the Lp3 BGC	This work
pIJ12819	pIJ10257 with the complete Lp2 BGC	This work

### Construction of pIJ12815.

A 2.6-kb DNA fragment containing the BGC for Lp3 was PCR amplified with primers JP127_NdeI (catATGGAACCCCAGATGACTGAG; introduced enzyme restriction sites here and below are underlined, and nucleotides not present in the genome sequence of *S. leeuwenhoekii* are lowercase) and JP128_PacI (ttaattaaCGTGTCGACCGGTGTCAGG) and genomic DNA from S. leeuwenhoekii C34^T^ as the template. The PCR product was first cloned into SmaI-digested pBluescript II KS(+) and the sequence verified by Sanger sequencing with universal primers M13F-24mer (CGCCAGGGTTTTCCCAGTCACGAC) and M13R-22mer (TCACACAGGAAACAGCTATGAC). The verified insertion from one clone was excised with NdeI and PacI and cloned in pIJ10257 digested with the same enzymes, generating pIJ12815. pIJ12815 was introduced into the heterologous *Streptomyces* expression hosts by conjugation selecting for hygromycin-resistant integrants (24).

### Construction of pIJ12819.

A 2.8-kb DNA fragment containing the biosynthetic genes for Lp2 (*leeCEBA*) was PCR amplified with primers JP138_NdeI (aacatATGGAACCCTGCGTCCCG) and JP139_HindIII (aaaagcttAGACCCTCATCCGCGCAATG) and genomic DNA from S. leeuwenhoekii C34^T^ as the template. Independently, a 3.8-kb DNA fragment containing the precursor peptide and transport genes for Lp2 (*leeADF*) was PCR amplified with primers JP143_AvrII (aacctaggGCGGGGTGACCTGAGGTGGATG) and JP144_NdeI (aacatATGGAGCACGACGAGAAGACG) and genomic DNA from S. leeuwenhoekii C34^T^ as the template. In both cases, the PCR product was first cloned into SmaI-digested pBluescript II KS(+) and the sequence verified by Sanger sequencing with universal primers M13F-24mer (CGCCAGGGTTTTCCCAGTCACGAC) and M13R-22mer (TCACACAGGAAACAGCTATGAC). The *leeADF* fragment was excised by digestion with BsiWI (internal to *leeA*) and NotI (provided by the vector polylinker) and ligated to the pBluescript II KS(+) derivative containing *leeCEBA* cut with the same enzymes, reconstructing the complete Lp2 *leeCEBADF* BGC (see Fig. S5 in the supplemental material). The insertion from one clone was excised with NdeI and AvrII and cloned in pIJ10257 digested with the same enzymes, generating pIJ12819. pIJ12819 was introduced into the heterologous *Streptomyces* expression hosts by conjugation selecting for hygromycin-resistant integrants (24).

### Large-scale production and purification of leepeptin.

S. coelicolor M1623 (S. coelicolor M1152 containing pIJ12819) was cultured in 25 ml of production medium (12.5 ml tryptone soya broth [TSB; Difco]) plus 12.5 ml yeast extract-malt extract (YEME) broth (YEME composition per liter, 3 g yeast extract, 3 g malt extract, 5 g bacteriological peptone, 10 g glucose, and 170 g sucrose) in a 250-ml Erlenmeyer flask for 8 days. An aliquot of the culture was used to inoculate 150 ml TSB/YEME (50:50) in a 500-ml Erlenmeyer flask and grown for 7 days at 30°C and with shaking at 200 rpm. This culture was used to inoculate five 2-liter baffled Erlenmeyer flasks containing 300 ml TSB/YEME (50:50) and incubated for 7 days at 30°C and with shaking at 200 rpm. The purity of the culture was verified at each step by streaking out an aliquot of the culture on tryptic soy agar (Becton, Dickinson). The cultures were harvested at 4,000 rpm for 10 min and the supernatant fractions pooled and mixed with 6% (wt/vol) Diaion HP-20 resin beads (Mitsubishi) and left overnight. The resin beads were recovered by filtering the solution through glass wool and then soaked in methanol. The methanol fraction was filtered through filter paper in a vacuum system. The eluent was concentrated at 40°C at reduced pressure, and the resulting solution was fractionated through an ion exchange column (Strata SAX) (55-μm pore size, 70 Å, 500 mg/3 ml) using 10% of the packing weight as the loading fraction. The column was equilibrated with 20 column volumes of water before the sample was loaded and washed with 10 column volumes (30 ml) of different mixtures of methanol-water solutions i to v as follows: (i) water; (ii) 75% water and 25% methanol; (iii) 50% water and 50% methanol; (iv) 25% water and 75% methanol; (iv) 100% methanol; (v) 100% methanol and 0.1% trifluoroacetic acid (TFA). An aliquot of each fraction was concentrated and analyzed by liquid chromatography-mass spectrometry (LC-MS), and the peptide was detected with highest intensity in the fraction containing 75% methanol. This fraction was further concentrated and subjected to semipreparative high-performance liquid chromatography (HPLC; Agilent 1200) through a C_18_ column (Waters SunFire) (5-μm particle size, 100-Å pore size, 250 by 10 mm) connected to a binary pump and a photodiode array detector set at 280 nm. Solvents used were Milli-Q water–0.1% (vol/vol) formic acid (solvent A) and methanol–0.1% (vol/vol) formic acid (solvent B). A gradient was set from 0% to 100% solvent B in 30 min and then 100% solvent B for 5 min, giving a total run time of 35 min, with a flow rate of 2 ml/min. The fraction containing the peptide eluted at around 22 to 23 min. The purified peptide was obtained in a volume of 3 mg, and the purity of the peptide was assessed by LC-MS (Fig. S10).

### Mass spectrometric analyses.

Values corresponding to the ions expected from peptide fragmentation by tandem mass spectrometry (MS/MS) were calculated with Fragment Ion Calculator software (http://db.systemsbiology.net:8080/proteomicsToolkit/FragIonServlet.html). For the calculation of A and B ions, 18.0106 Da was deducted to account for the loss of a water molecule during cyclization. For MALDI-ToF analyses, the peptide samples were spotted onto a prespotted AnchorChip MALDI target plate (α-cyano-4-hydroxycinnamic acid matrix; Bruker Daltonics, Coventry, United Kingdom), and the spots were washed briefly with 10 mM ammonium phosphate–0.1% TFA according to the manufacturer’s instructions. After drying, the samples were analyzed using MALDI-ToF and an Ultraflex TOF/TOF mass spectrometer (Bruker). The instrument was calibrated using the prespotted standards (ca. 200 laser shots). Samples were analyzed using a method optimized for peptide analysis, and spectra were summed from ca. 30 × 15 laser shots. The data were processed in FlexAnalysis (Bruker). Several liquid chromatography-mass spectrometry instruments and methods were employed. LC-IT-ToF MS methods were performed as previously published (28); briefly, samples were analyzed on a Shimadzu NexeraX2 LC instrument fitted with a Prominence photo diode array detector and with an LC-IT-ToF mass spectrometer set for positive-ion-mode detection; samples were injected in a Kinetex XB-C_18_ column (part no. 00B-4496-AN; Phenomenex, USA) (2.6-μm particle size, 100-Å pore size, 50 by 2.10 mm) and eluted with a gradient of 0.1% formic acid–water–methanol (2% to 100% methanol) over 9.5 min, at a flow rate of 0.6 ml per min; data acquisition and analysis were performed with LCMSsolution version 3 (Shimadzu). High-resolution mass spectra were acquired by LC-HRMS on a Synapt G2 mass spectrometer equipped with an Acquity ultraperformance liquid chromatograph (UPLC; Waters, Wilmslow, United Kingdom). Aliquots of the samples were injected onto an Acquity UPLC ethylene bridged hybrid (BEH) C_18_ column (Waters) (1.7-μm particle size, 100-Å pore size, 1 by 100 mm) and eluted with a gradient of (solvent B) acetonitrile–0.1% formic acid mixed with (solvent A) water–0.1% formic acid with a flow rate of 0.4 ml/min at 45°C. The concentration of solvent B was kept at 3% for 1 min followed by a gradient up to 50% solvent B in 12 min. MS data were collected with the following parameters: resolution mode; positive-ion mode; scan time, 1 s; mass range, *m*/*z* 50 to 2,000 (calibrated with sodium formate); capillary voltage, 3.0 kV; cone voltage, 40 V; source temperature, 150°C; desolvation temperature, 500°C. Leu-enkephalin peptide was used to generate a lock-mass calibration with 556.2766 *m*/*z* measured every 10 s during the run. For MS/MS fragmentation, a data directed analysis (DDA) method was used with the following parameters: precursor selection from inclusion list only (807.5; 1,170.5), MS/MS threshold, 7,000; scan time, 5 s; no dynamic exclusion; collision energy (CE) ramped between 20 and 30 at low mass (*m*/*z* 50) and 30 and 60 at high mass (*m*/*z* 2,000). HRMS was also performed on an Agilent Technology 1290 Infinity II system fitted with a Kinetex C_18_ column (part no. 00D-4462-AN; Phenomenex Inc.) (2.6-μm particle size, 100-Å pore size, 100 by 2.1 mm), which was kept at 40°C. The system was coupled to a quadrupole time of flight (QTOF) maXis II mass spectrometer (Bruker Inc.). For liquid chromatography, a gradient of 95% Milli-Q water to 100% solvent B (acetonitrile–0.1% formic acid) was used over 10 min with a 5-μl injection volume. Mass spectrometry was deployed in positive-ionization mode with a mass/charge range of 50 to 2,000. MS/MS fragmentation of purified leepeptin was obtained using direct injection into a LTQ XL Orbitrap mass spectrometer using 35% relative collision energy. Marfey’s analysis was carried out using Marfey’s FDLA [Nα-(2,4-dinitro-5-fluorophenyl)-l-leucinylamide] standard (Sigma-Aldrich) and the protocol described in reference 4.

### Nuclear magnetic resonance.

All 1D and 2D NMR experiments were performed using a Bruker Avance III HD 600-MHz spectrometer (Ascend 14.1 Tesla) with a Prodigy TCI cryoprobe at 25°C.

## Supplementary Material

Supplemental file 1
