# Supplementary material for: Heterologous Expression of a Cryptic Gene Cluster from Streptomyces leeuwenhoekii C34T Yields a Novel Lasso Peptide, Leepeptin
Source: Appl Environ Microbiol. 2019 Nov 14;85(23):e01752-19. doi: 10.1128/AEM.01752-19 (PMC6856326; doi:10.1128/AEM.01752-19)
Supplement: Supplemental file 1 [file AEM.01752-19-s0001.pdf]

Supplemental Material to:

Heterologous expression of a cryptic gene cluster from *Streptomyces leeuwenhoekii* C34^T yields a novel lasso peptide, leepeptin

Juan Pablo Gomez-Escribano,[a] Jean Franco Castro,[a,b,c*] Valeria Razmilic,[a,b] Scott A. Jarmusch,[c] Gerhard Saalbach,[a] Rainer Ebel,[c] Marcel Jaspars,[c] Barbara Andrews,[b] Juan A. Asenjo,[b] Mervyn J. Bibb [a]#

[a] Department of Molecular Microbiology, John Innes Centre, Norwich Research Park, Norwich, United Kingdom

[b] Centre for Biotechnology and Bioengineering (CeBiB), Department of Chemical Engineering and Biotechnology, Universidad de Chile, Santiago, Chile

[c] Marine Biodiscovery Centre, Department of Chemistry, University of Aberdeen, Old Aberdeen, United Kingdom

Running title: Leepeptin, a novel lasso peptide

# Address correspondence to Mervyn J. Bibb, mervyn.bibb@jic.ac.uk

* Present address: Instituto de Investigaciones Agropecuarias (INIA) Quilamapu, Chillán, Chile.

J.F.C., V.R. and S.A.J. contributed equally to this work.

FIG S1 MALDI-ToF analysis of *S. leeuwenhoekii* C34^T cultures grown in different media showing the detection of Lp3-related ions (1614.832 *m/z* for $[M+H]^+$, 1636.820 *m/z* for $[M+Na]^+$).

Spectrum of Lp3 from a culture supernatant subsequently confirmed to contain the molecule by LC/Tandem-HRMS as reference

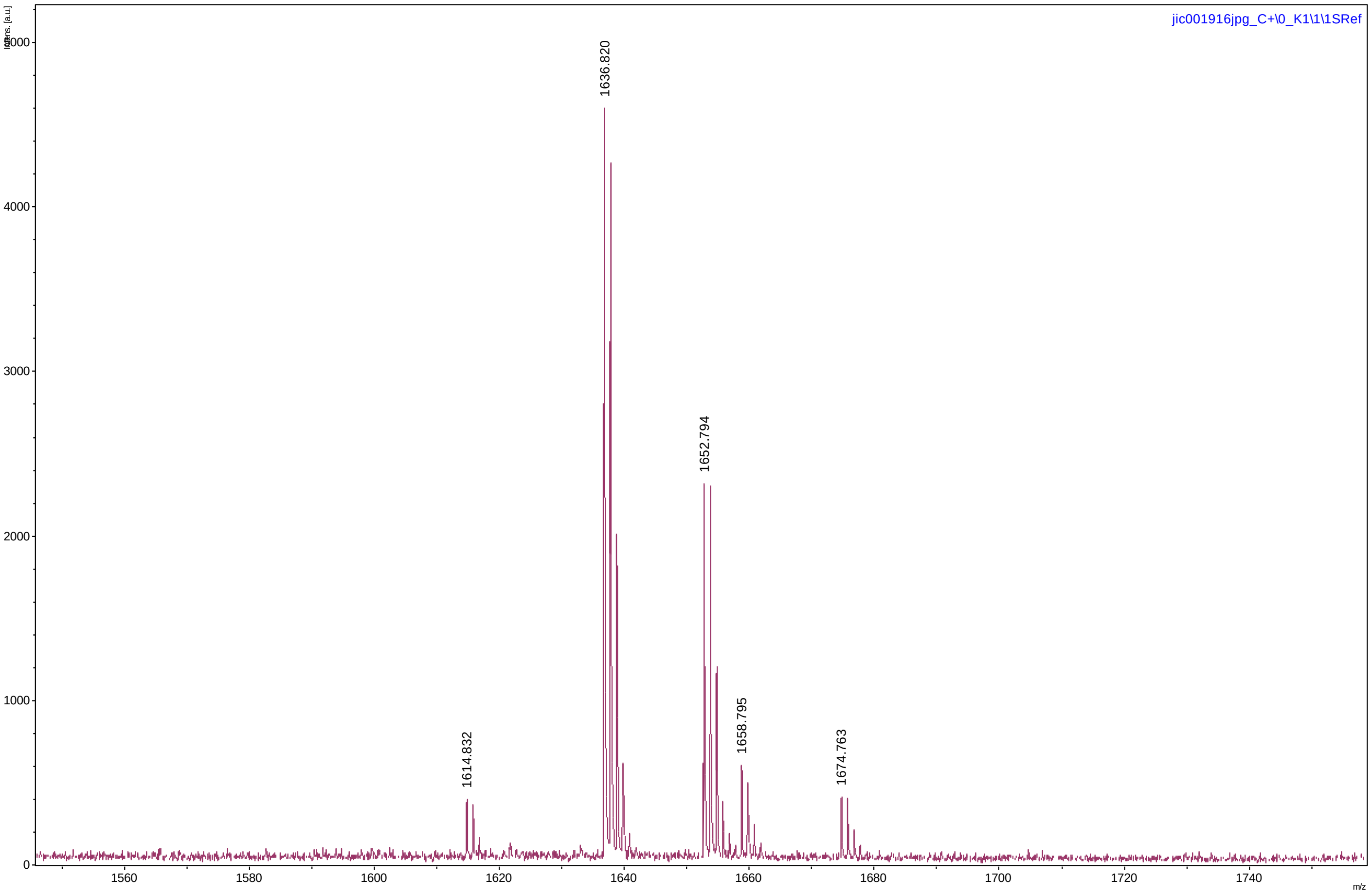

S. leeuwenhoekii C34^T grown in LPM for 3d

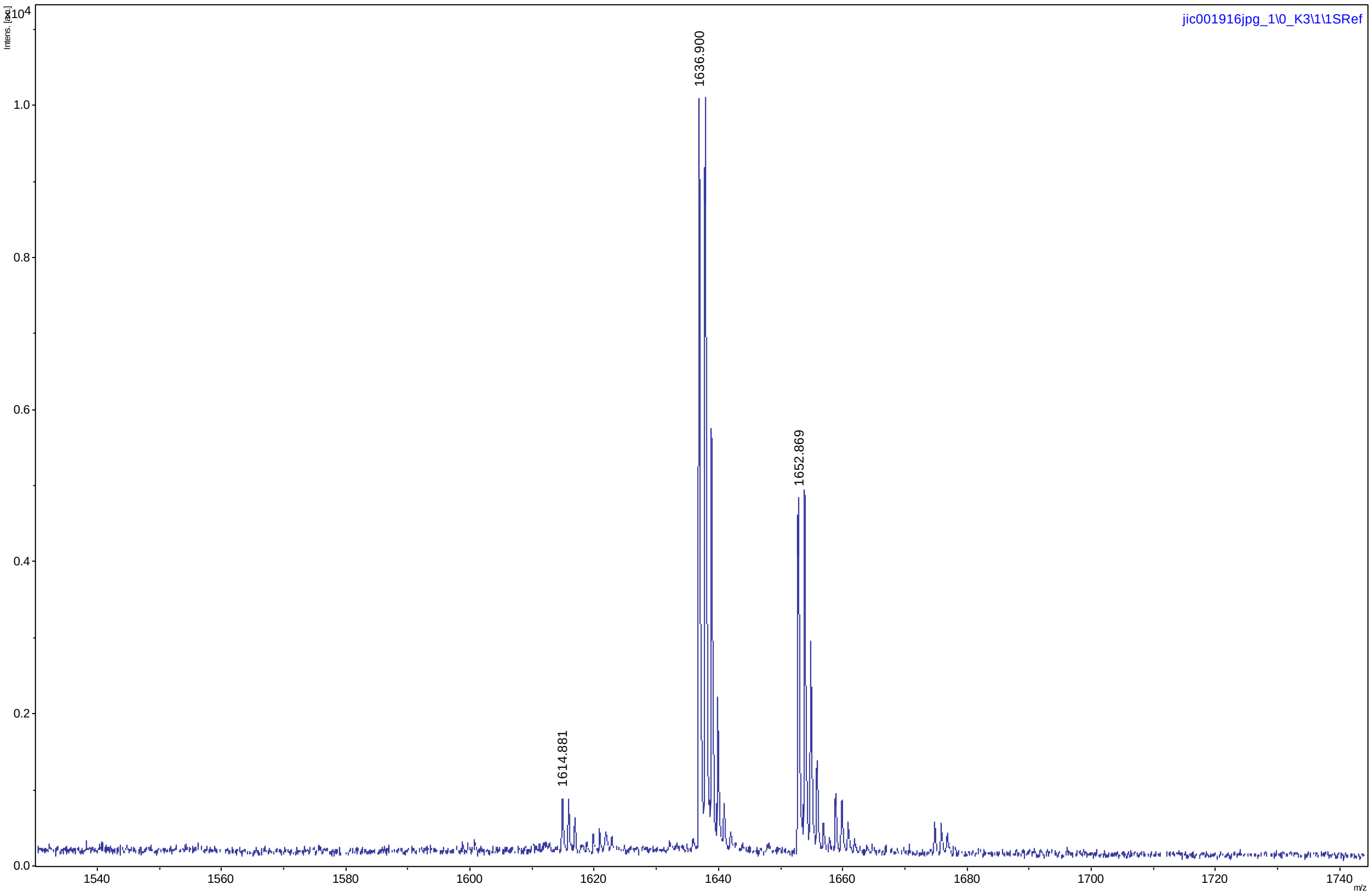

30 *S. leeuwenhoekii* C34[T] grown in LS for 3d

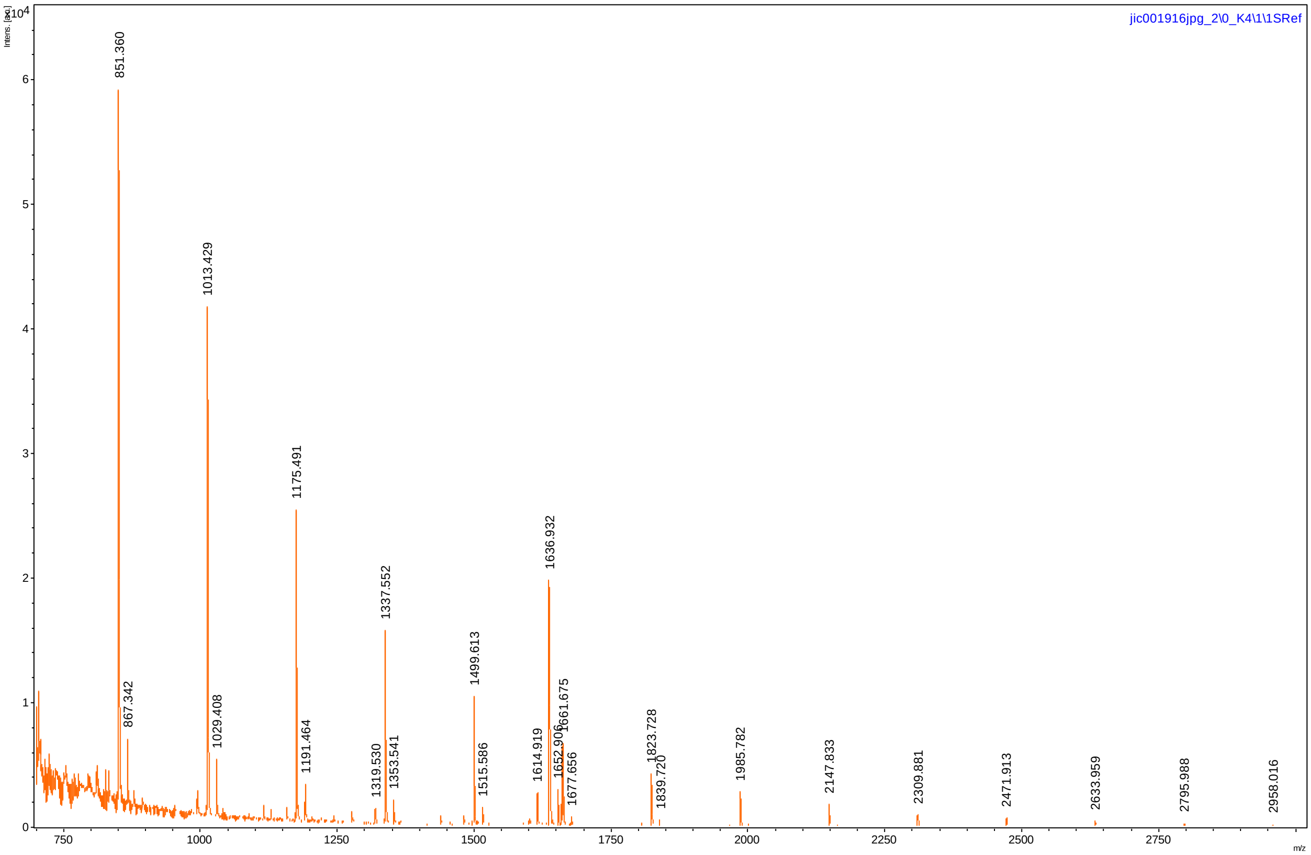

31

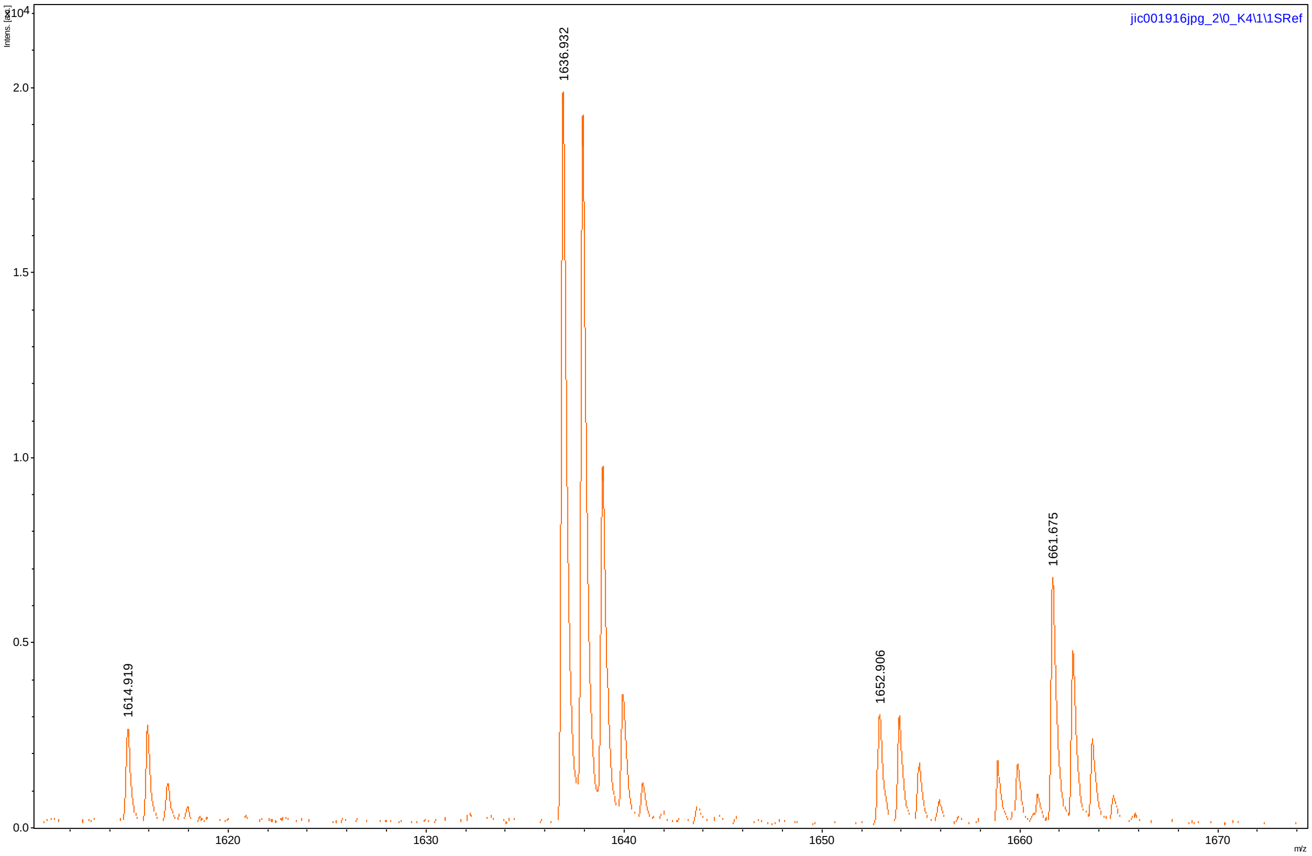

32

S. leeuwenhoekii C34^{T} grown in modified ISP2 for 3d

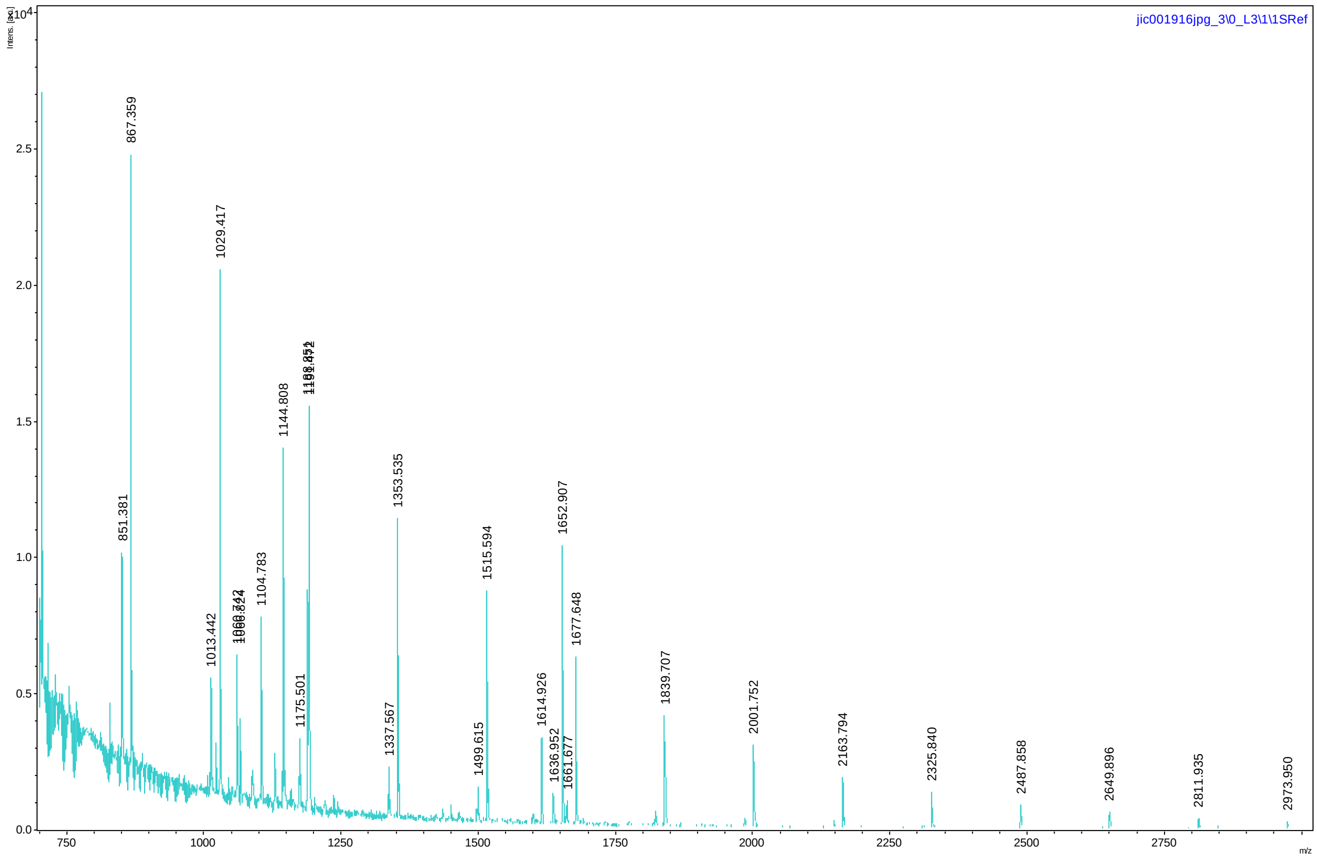

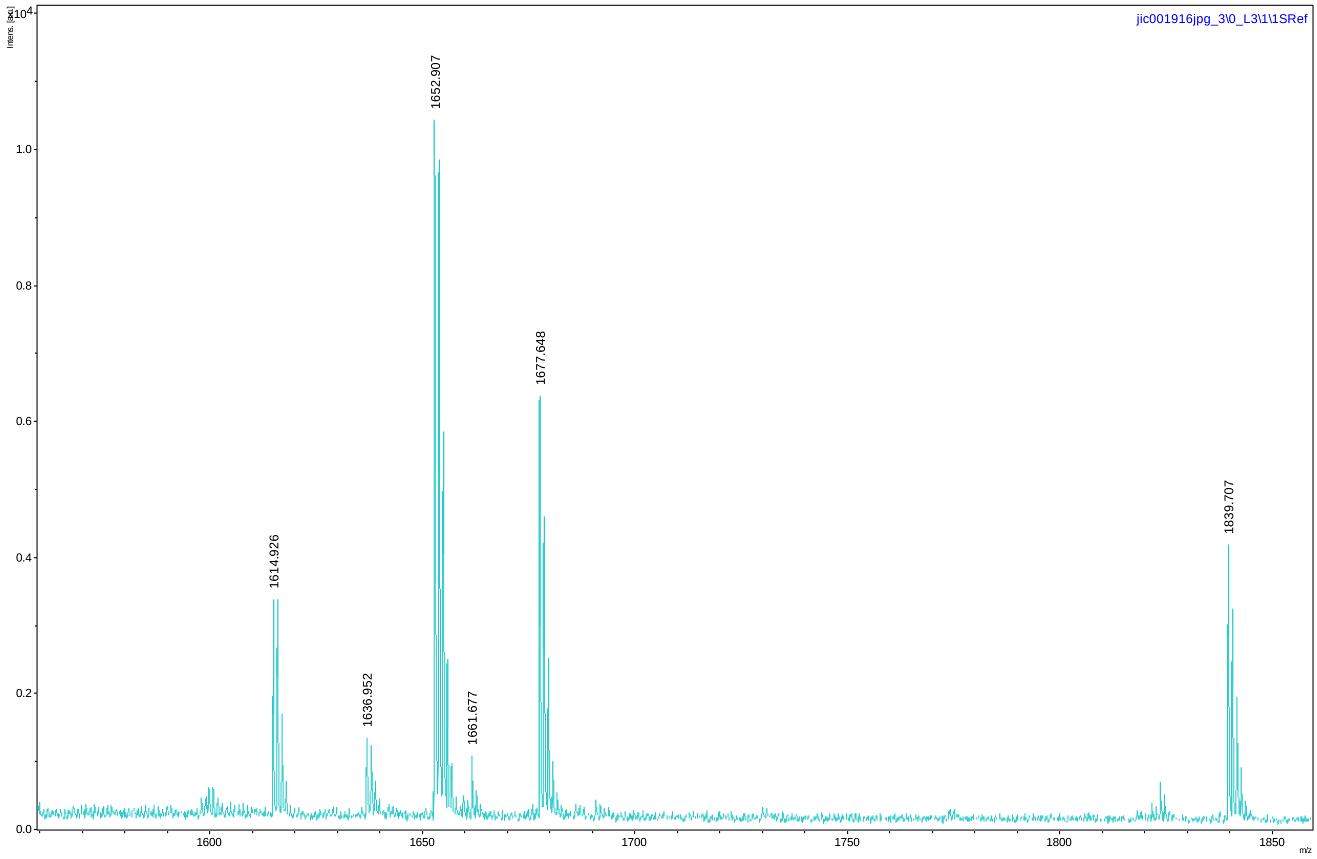

S. leeuwenhoekii C34[T] grown in R3 for 3d

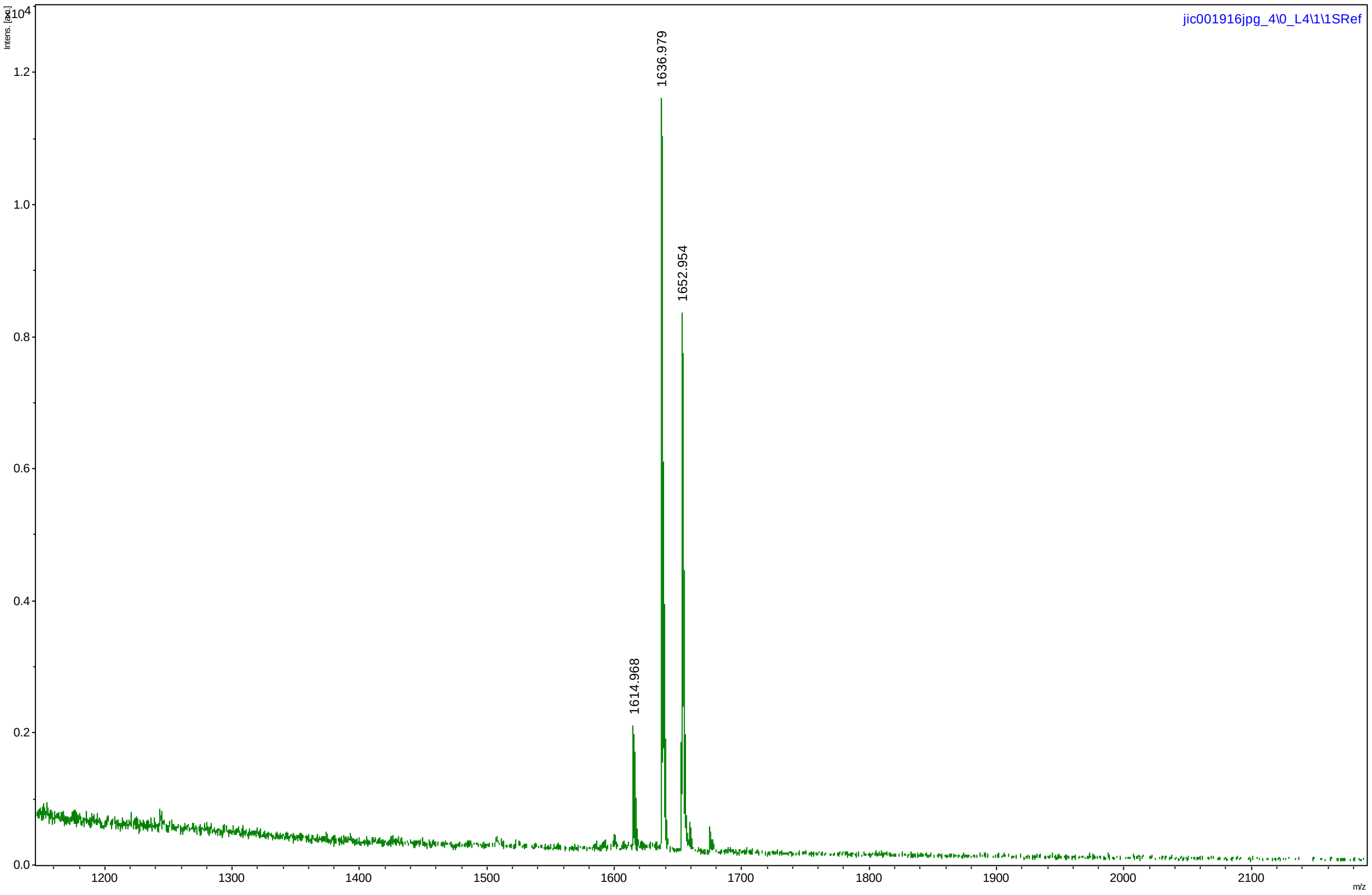

S. leeuwenhoekii C34^{T} grown in TY for 3d

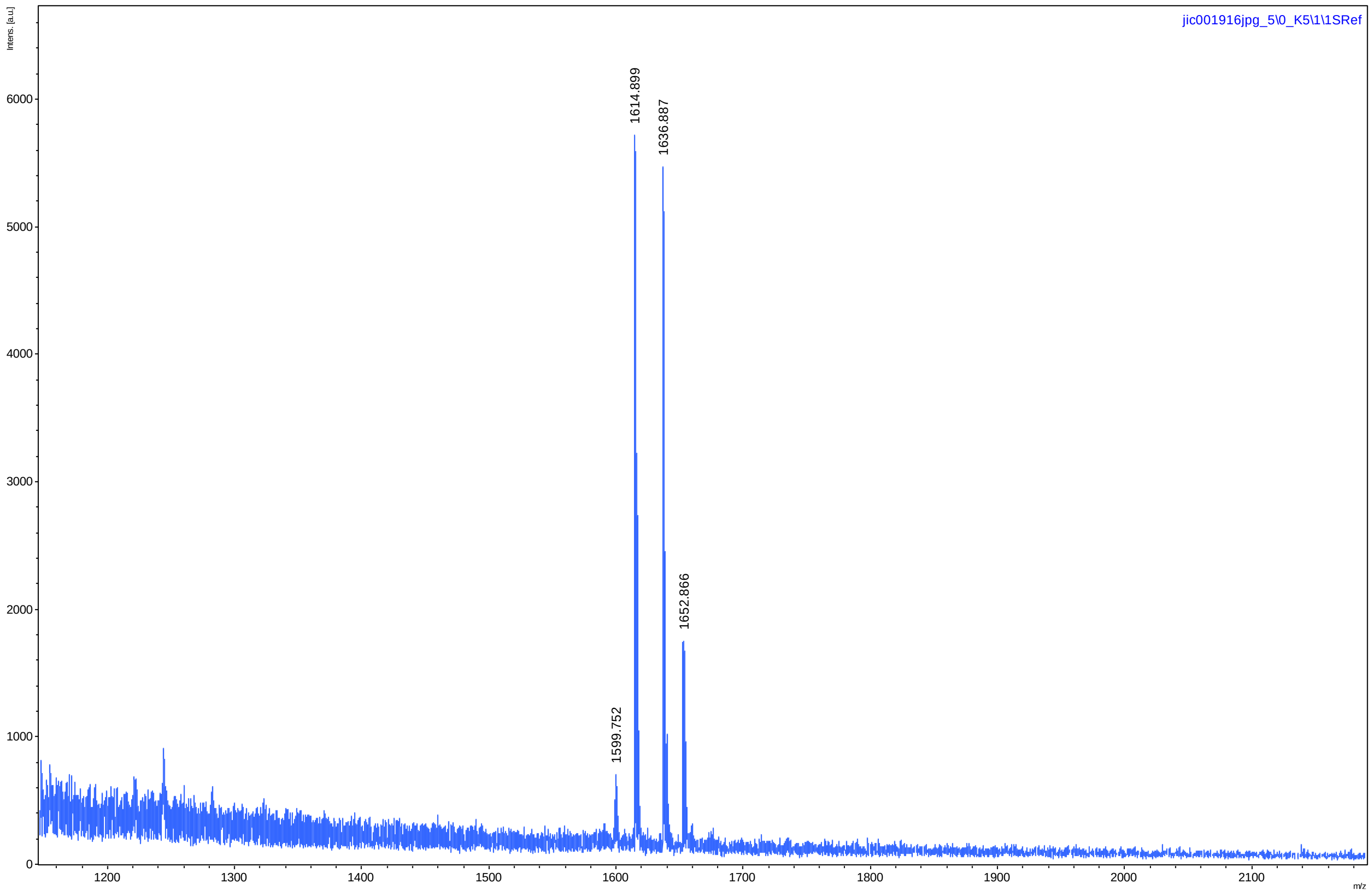

FIG S2 Predicted structure of Lp3 and the ions expected after fragmentation of the molecule by LC/Tandem-HRMS. An example of a deconvoluted spectrum from *S. leeuwenhoekii* C34^{T} obtained on a Waters Synapt G2Si with QToF MS detector is shown. The table of expected ions was calculated for the linear peptide after subtraction of a water molecule from the b- and a- ions (using http://db.systemsbiology.net:8080/proteomicsToolkit/FragIonServlet.html).

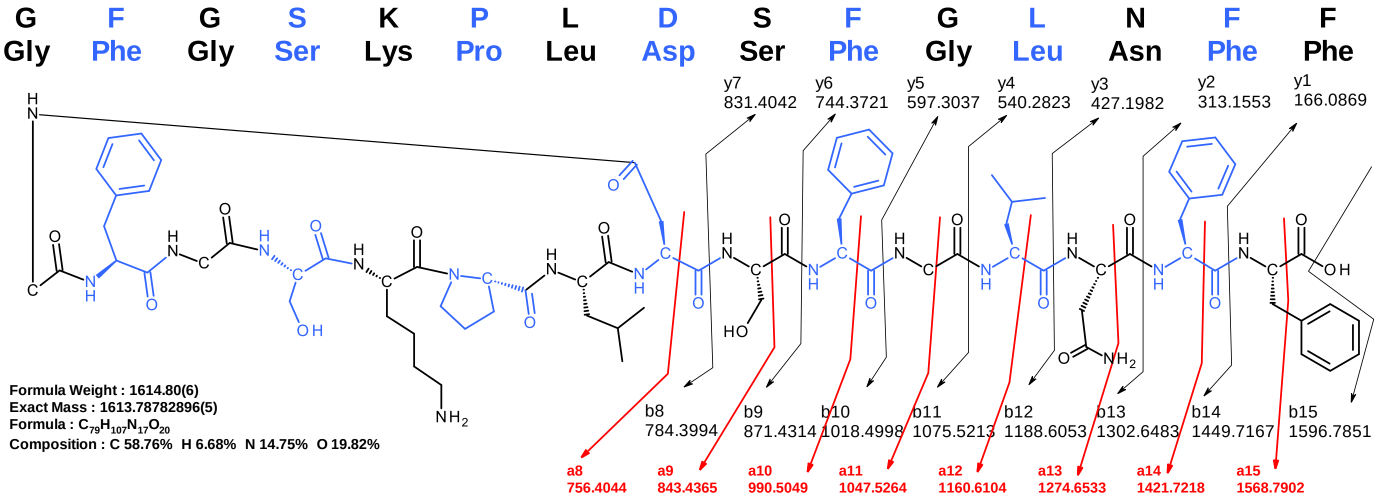

Seq	#	A-loop	B-loop	Y-loop	#
G	1	12.02386	40.01877	1632.806	15
F	2	159.0923	187.0872	1575.785	14
G	3	216.1137	244.1086	1428.716	13
S	4	303.1458	331.1407	1371.695	12
K	5	431.2407	459.2356	1284.663	11
P	6	528.2935	556.2884	1156.568	10
L	7	641.3775	669.3725	1059.515	9
D	8	756.4045	784.3994	946.4311	8
S	9	843.4365	871.4314	831.4042	7
F	10	990.5049	1018.5	744.3721	6
G	11	1047.526	1075.521	597.3037	5
L	12	1160.61	1188.605	540.2823	4
N	13	1274.653	1302.648	427.1982	3
F	14	1421.722	1449.717	313.1553	2
F	15	1568.79	1596.785	166.0869	1

In boxes: ions which we expected to detect based on cyclisation between G1 and D8

Filled in blue: ions with a predicted m/z that were observed in samples from *S. leeuwenhoekii* C34^{T} and the heterologous producers

1614.796 *m/z* is the monoisotopic $[M+H]^{+}$ for the loop-containing peptide and so is always detected

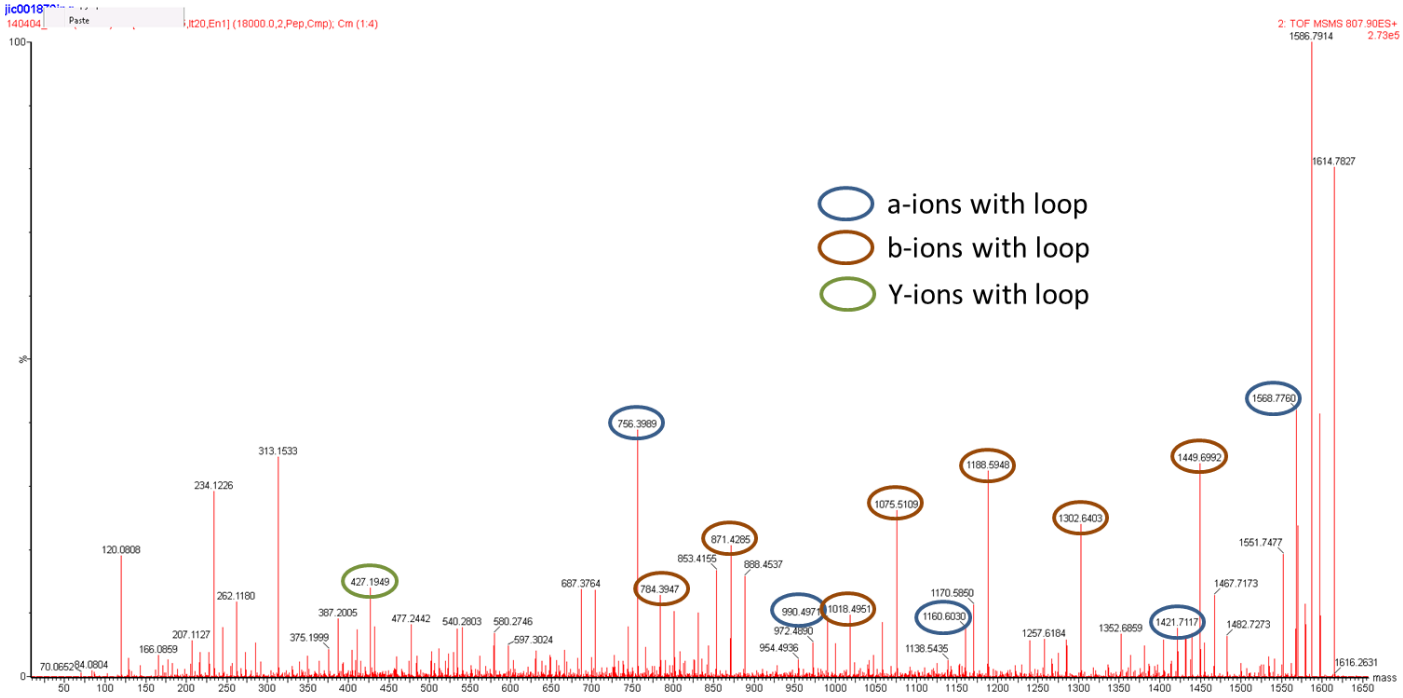
jic0018
Paste
,It20,En1] (18000.0,2,Pep,Cmp); Cm (1:4)
2: TOF MSMS 807.90ES+
2.73e5
a-ions with loop
b-ions with loop
Y-ions with loop
1586.7914
1614.7827
1568.7760
756.3989
313.1533
234.1226
120.0808
1449.6992
1188.5948
1075.5109
1302.6403
871.4285
853.4155
888.4537
1551.7477
427.1949
784.3947
687.3764
262.1180
1467.7173
1170.5850
990.4971
1018.4951
1160.6030
1421.7117
1482.7273
387.2005
477.2442
540.2803
580.2746
207.1127
375.1999
597.3024
972.4890
1257.6184
1352.6859
166.0859
954.4936
1138.5435
70.0652
84.0804
1616.2631
%
mass

FIG S3 Homologues of Lp3. Leader sequences are shown in blue and core peptide sequences in red

```
                                                                       Reference/Accession

Sungsanpin                                        GFGSKPIDSFGLSWL   1
                                                  |||||| |||||
Chaxapeptin (Lp3)     MTELQPEAYEAPSLIEVGEFSEDTL GFGSKPLDSFGLNFF   2 / WP_103143102
                        |  || || | ||||||||||||  |||| ||||||||
S. kanamyceticus  MESQVHEVLPEPYEPPALIEVGEFSEDTL GFGSRPLDSFGLNIR   WP_107099006
                  ||     |    || |||  ||||||||||  |||| |||||||| |
S. cinnamoneus    MEKMEEQVYADLYEAPALVEVGEFSEDTL GFGSKPLDSFGLNFR   WP_104531718
```

FIG S4 Detection of Lp3 by LC-IT-ToF MS after heterologous expression of the Lp3 BGC in different hosts (the identifier to the right of each chromatogram indicates the host strain and culture medium (TY) used; two independent ex-conjugants are shown for *S. coelicolor* M1152 + pIJ12185). Each of the panels displays an extracted ion chromatogram for the $[M+H]^+$ and $[M+2H]^{2+}$ ions with elution time shown on the x-axis and ion intensity on the y-axis. Singly charged ions are shown in red and doubly charged ions are shown in blue.

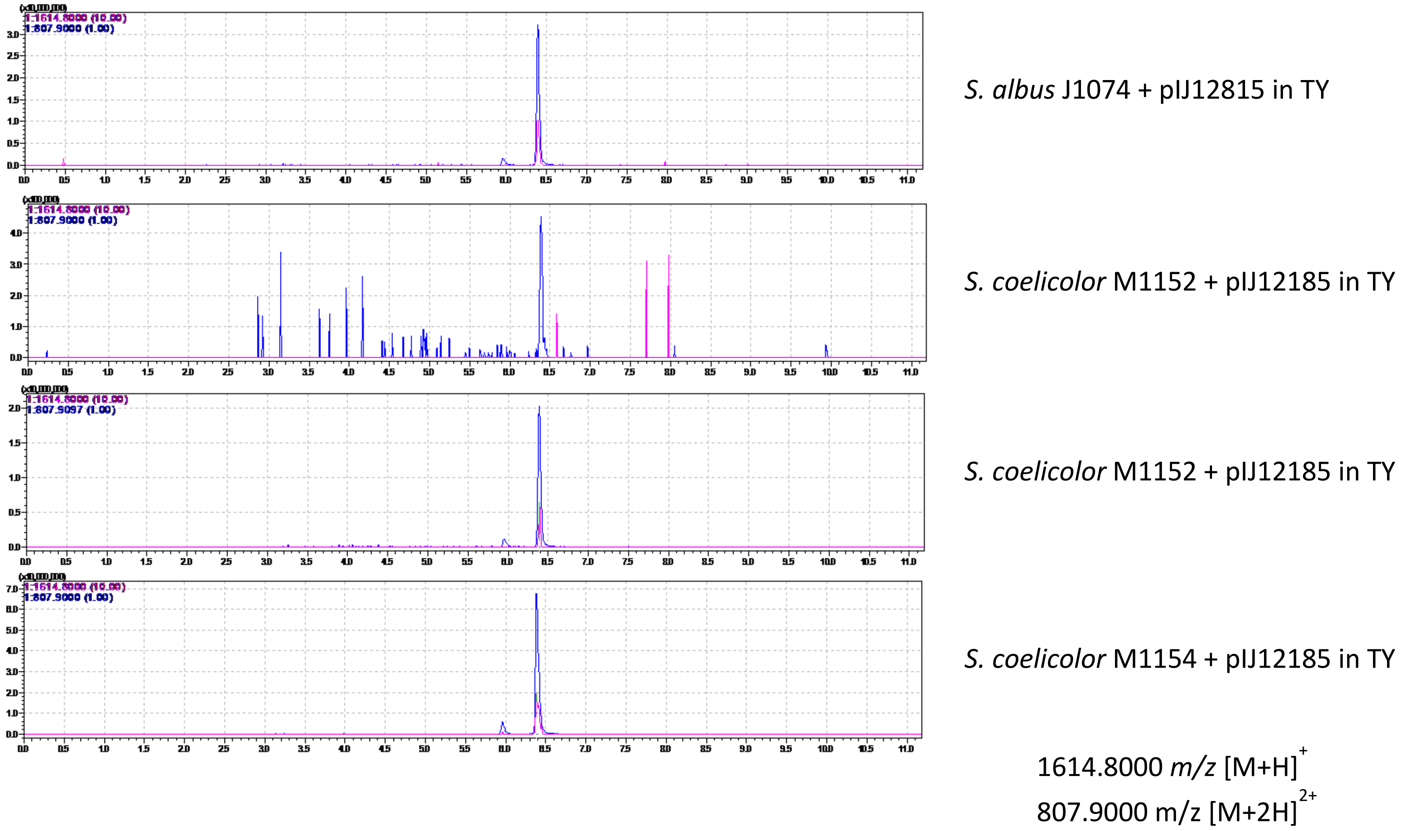

FIG S5 Strategy for construction of pIJ12819.

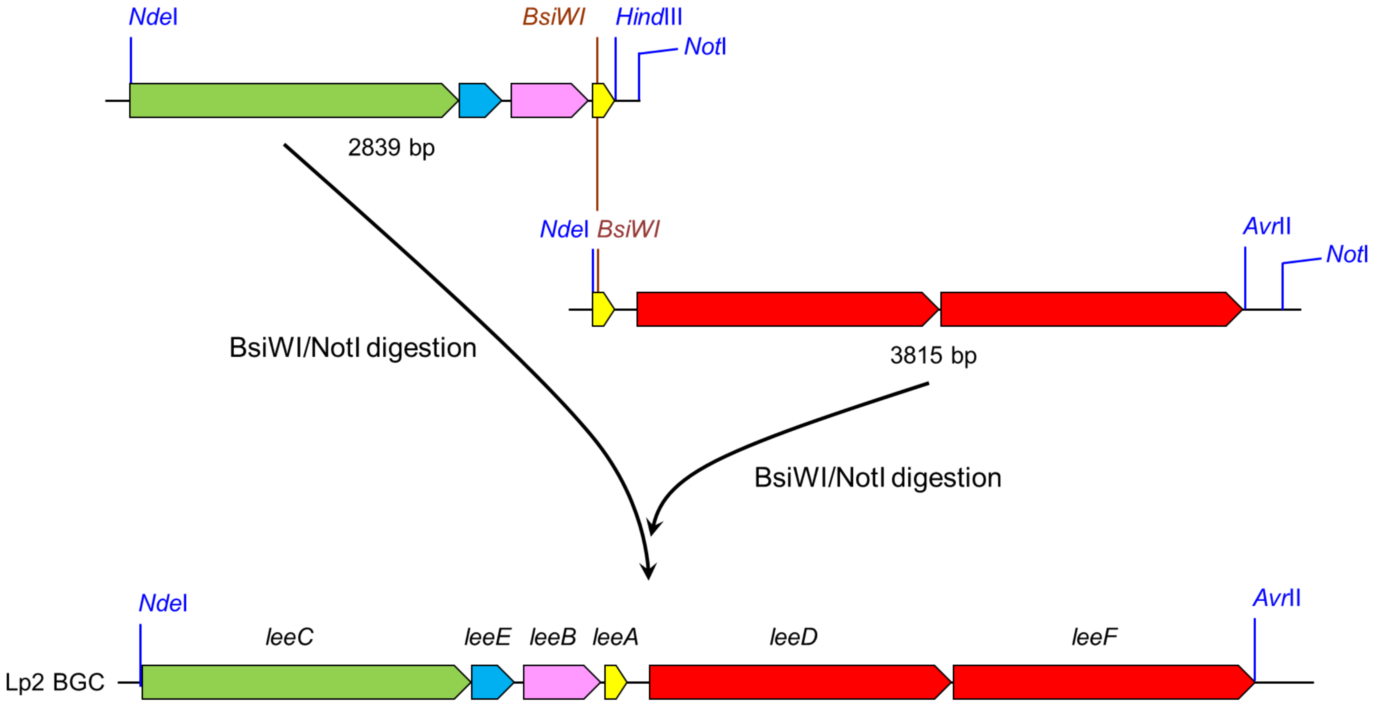

FIG S6 Initial LC/Tandem-HRMS analysis on a Synapt G2Si with QToF MS detector (Waters) of heterologously produced Lp2; screenshot of the data analysis software Masslinx after prediction of the peptide sequence which matches that expected from the nucleotide sequence of *leeA*.

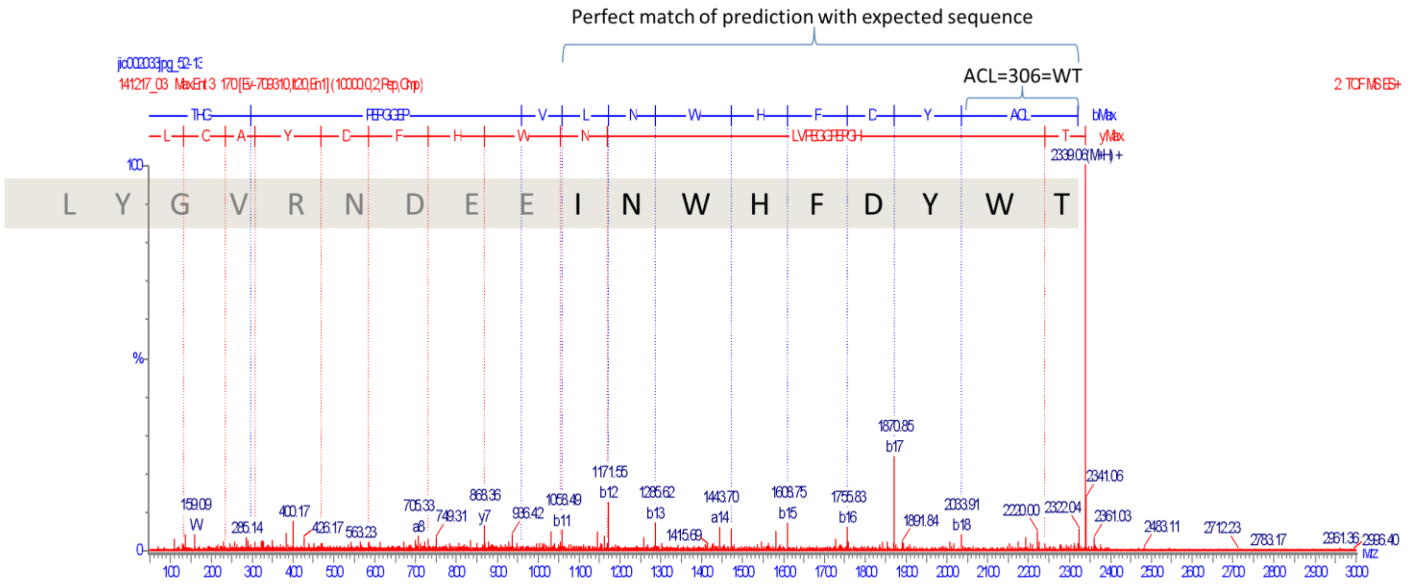

FIG S7 Deduction of the acidic residue of Lp2 involved in macrocyclisation with the N-terminal amino group by analysis of the deconvoluted spectra from LC/Tandem-HRMS on a Synapt G2Si with QToF MS detector (Waters). The images show the full and zoomed-in deconvoluted spectra. Ions 1030.4958, 1058.4921 and 1410.68 were readily apparent, while ions 901, 929 or 1539.65 were not detected, supporting the assignment of E8 as the acidic amino acid whose side chain participates in the cyclisation of the peptide.

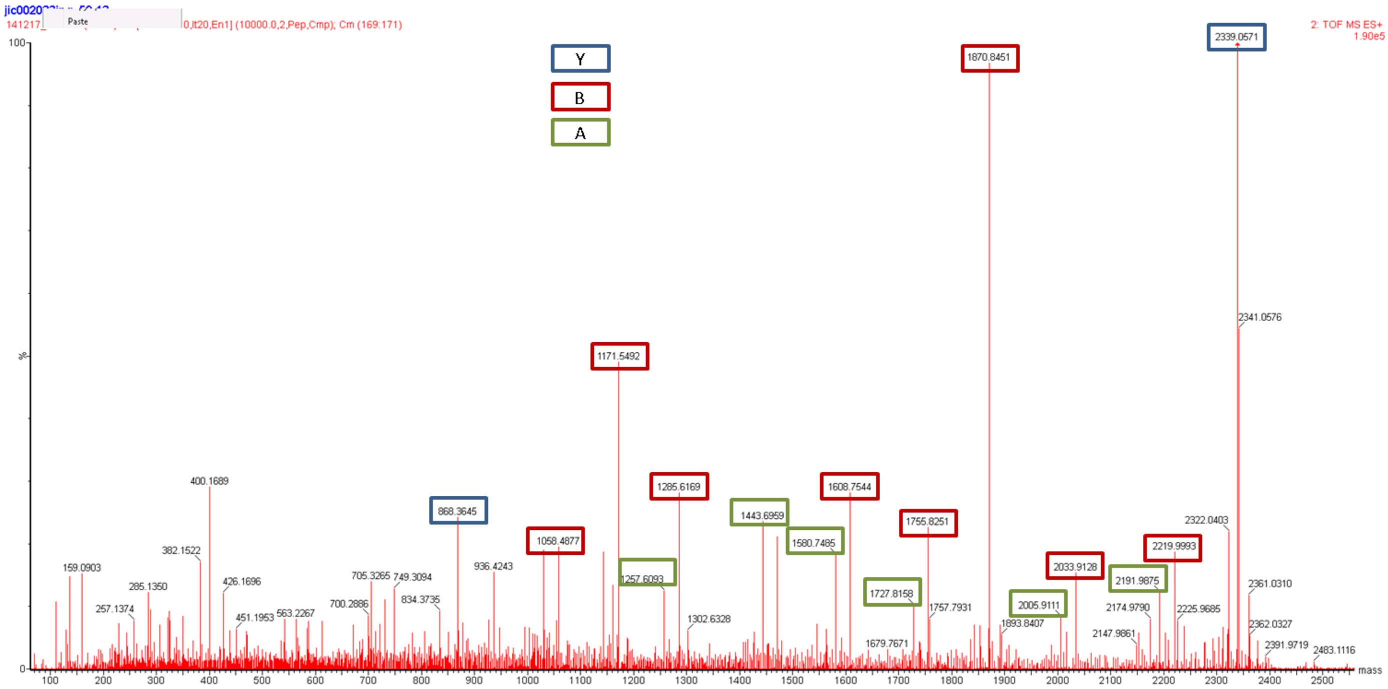

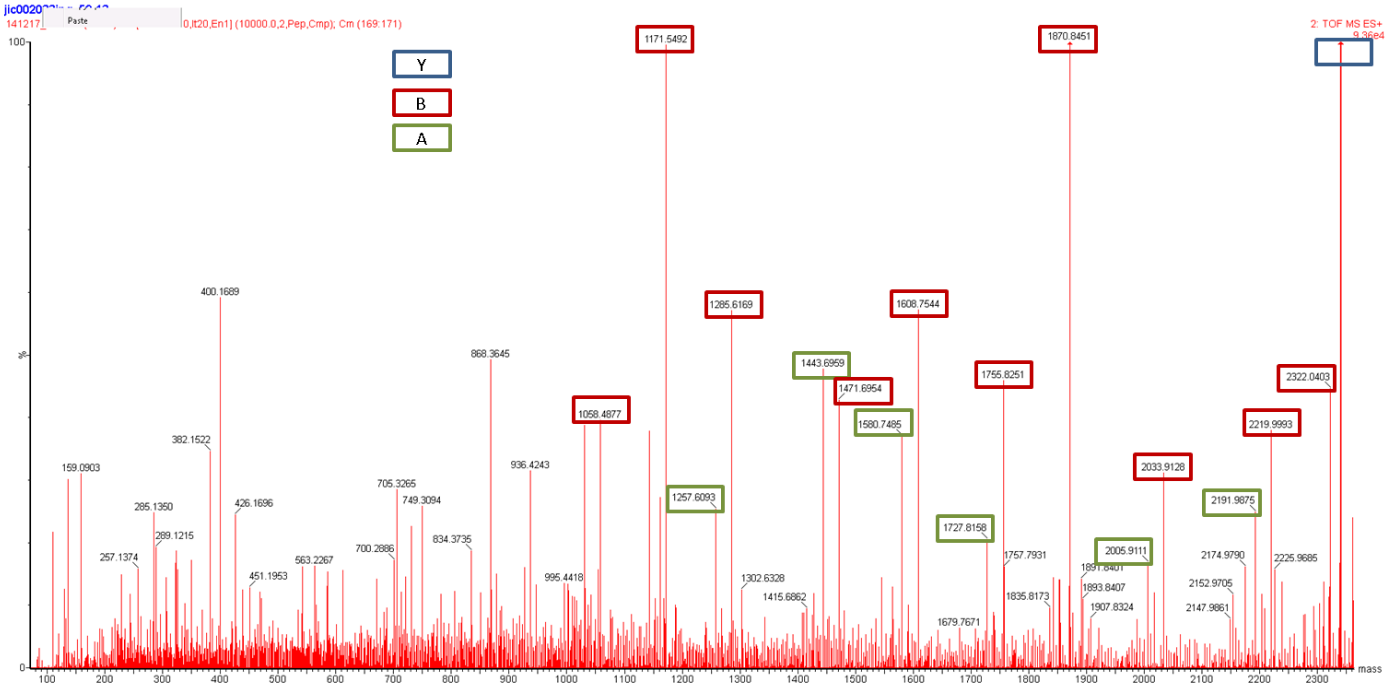

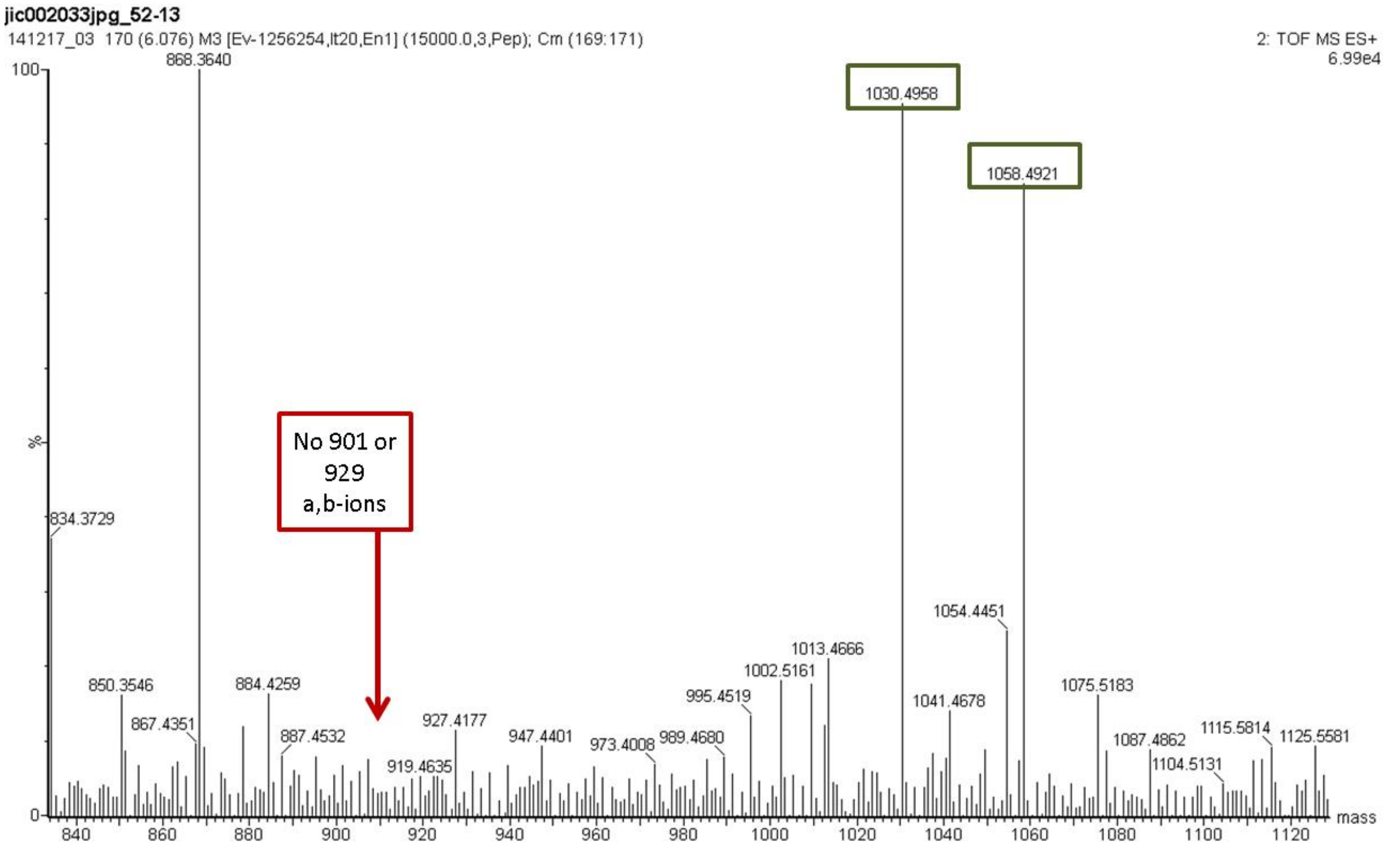
jic002033jpg_52-13
141217_03 170 (6.076) M3 [Ev-1256254,It20,En1] (15000.0,3,Pep); Cm (169:171)
2: TOF MS ES+
6.99e4
100
%
0
868.3640
1030.4958
1058.4921
No 901 or 929 a,b-ions
834.3729
850.3546
867.4351
884.4259
887.4532
919.4635
927.4177
947.4401
973.4008
989.4680
995.4519
1002.5161
1013.4666
1041.4678
1054.4451
1075.5183
1087.4862
1104.5131
1115.5814
1125.5581
mass
840 860 880 900 920 940 960 980 1000 1020 1040 1060 1080 1100 1120

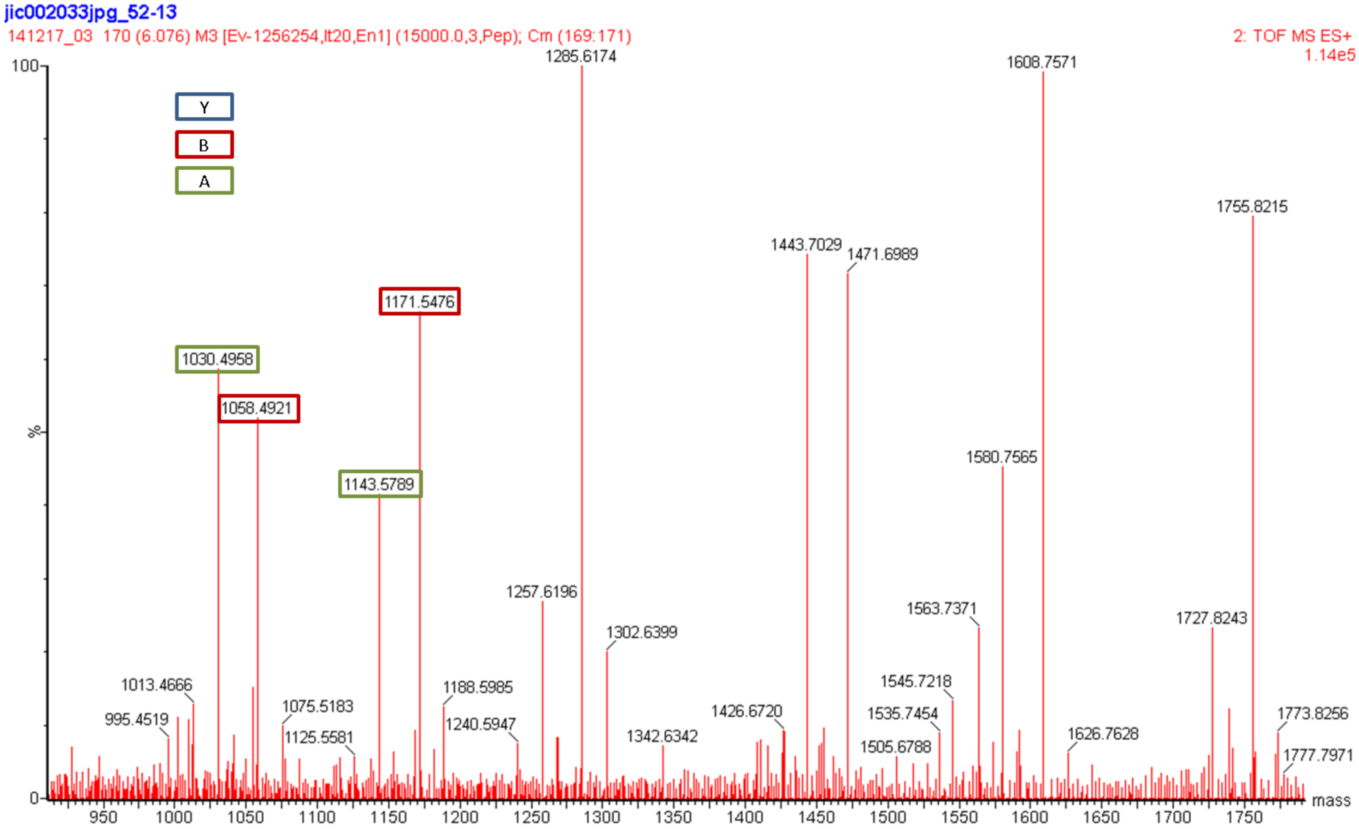
jic002033jpg_52-13
141217_03 170 (6.076) M3 [Ev-1256254,It20,En1] (15000.0,3,Pep); Cm (169:171)
2: TOF MS ES+
1.14e5
Y
B
A
100
%
0
1285.6174
1608.7571
1755.8215
1443.7029
1471.6989
1171.5476
1030.4958
1058.4921
1143.5789
1580.7565
1257.6196
1302.6399
1563.7371
1727.8243
1013.4666
995.4519
1075.5183
1125.5581
1188.5985
1240.5947
1342.6342
1426.6720
1545.7218
1535.7454
1505.6788
1626.7628
1773.8256
1777.7971
mass
950 1000 1050 1100 1150 1200 1250 1300 1350 1400 1450 1500 1550 1600 1650 1700 1750

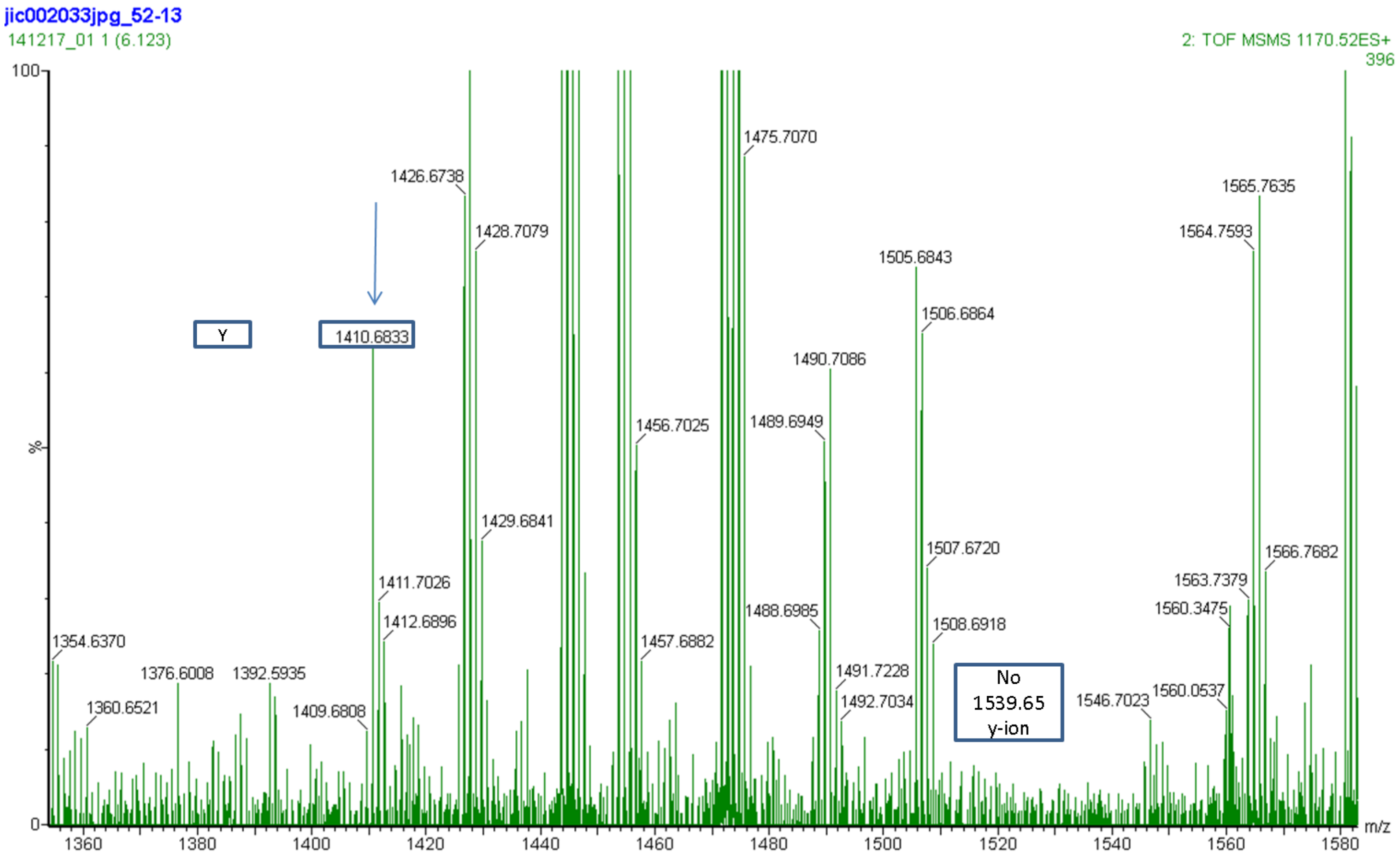
jic002033jpg_52-13
141217_01 1 (6.123)
2: TOF MSMS 1170.52ES+
396
100
%
0
m/z
Y
1410.6833
No
1539.65
y-ion
1354.6370
1360.6521
1376.6008
1392.5935
1409.6808
1411.7026
1412.6896
1426.6738
1428.7079
1429.6841
1456.7025
1457.6882
1475.7070
1488.6985
1489.6949
1490.7086
1491.7228
1492.7034
1505.6843
1506.6864
1507.6720
1508.6918
1546.7023
1560.0537
1560.3475
1563.7379
1564.7593
1565.7635
1566.7682
1360
1380
1400
1420
1440
1460
1480
1500
1520
1540
1560
1580

FIG S8 Tail-to-ring NOE correlations of plug residues (A) Phe14, (B) Tyr16, and (C) Trp17.

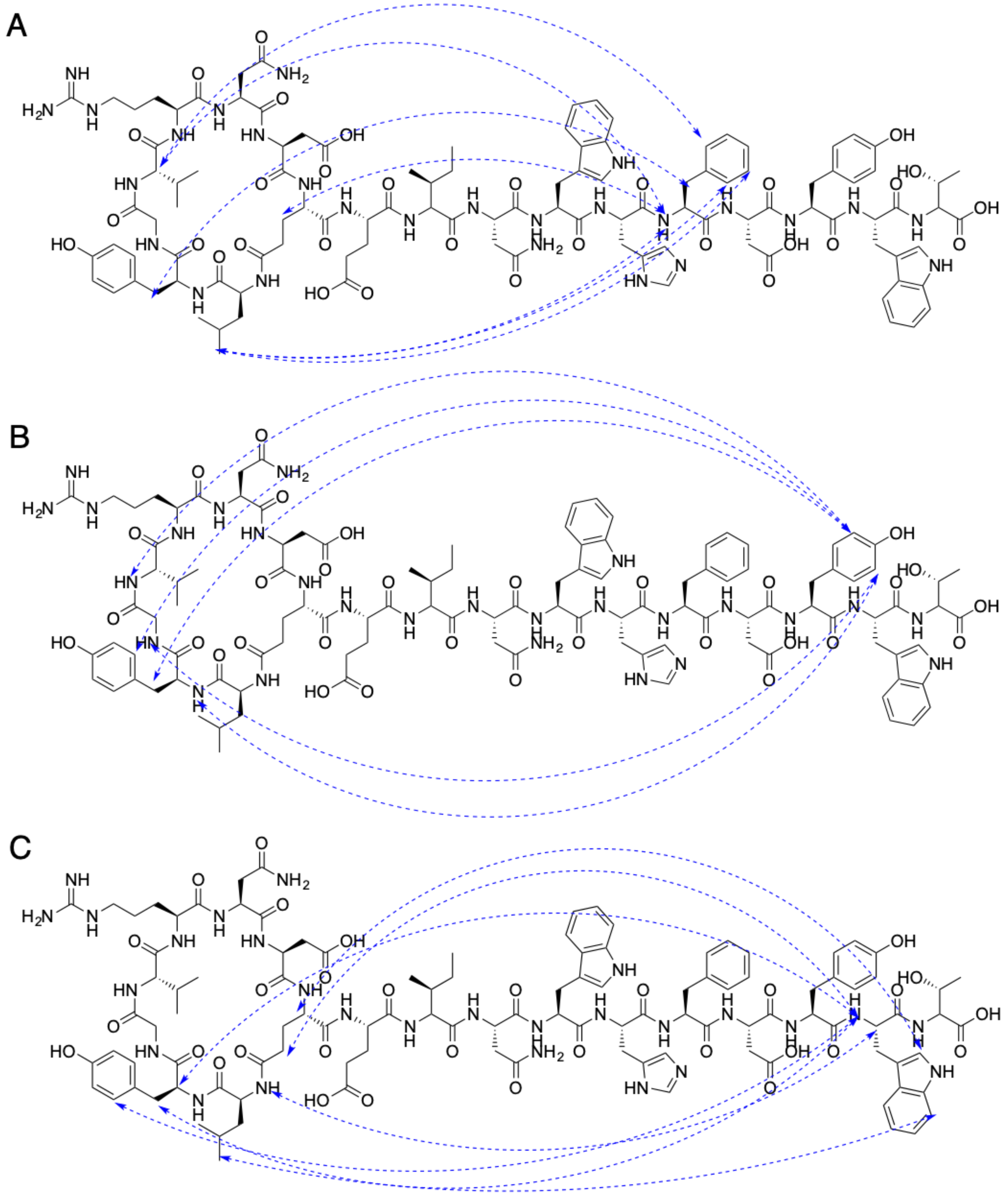

FIG S9 Sequence Similarity Network (SSN) analysis of the leepeptin sub-family of lasso peptides. Abbreviations: S.leu, *S. leeuwenhoekii* C34^{T}; S.sp L-9-10, *Streptomyces* sp. L-9-10; M.car, *M. carbonacea*; Am.xyl, *Amycolatopsis xylanica* and Ac.fib, *Actinomadura fibrosa*. Prior to analysis, the dataset of precursor peptides reported in reference 3 was supplemented with the four homologues identified by a blastP search of GenBank using the precursor peptide sequence of leepeptin as query sequence. The SSN was determined using the Enzyme Function Initiative-Enzyme Similarity Tool (EFI-EST) described in reference 4 using an E value of 1, and threshold score of 9. The SSN was displayed using Cytoscape (5). Only part of the network analysis that includes the leepeptin sub-family is shown.

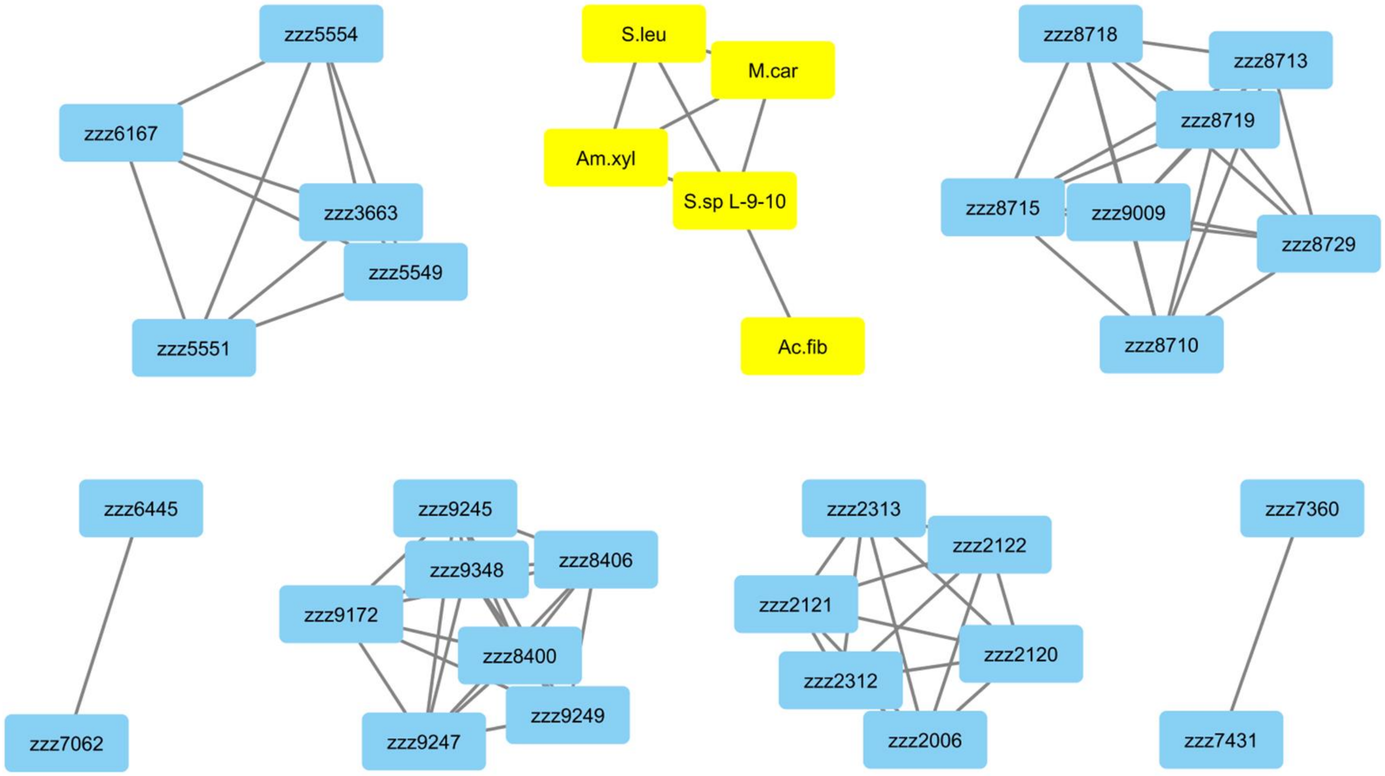

FIG S10 Total Ion Chromatograms (TIC) showing LC-MS traces of leepeptin (indicated by an asterisk with a retention time of 3.04 min). (A) TIC before purification; (B) TIC after purification.

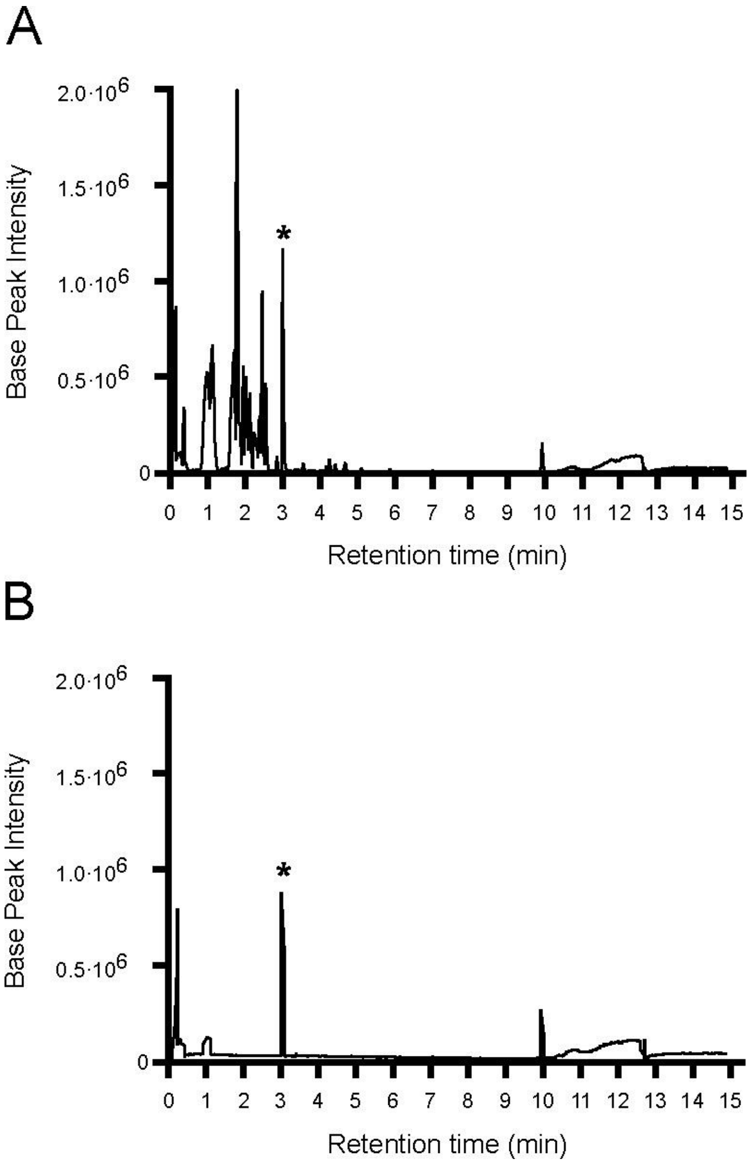

FIG S11 Numbered planar structure of leepeptin with atom numbers (see Table S2).

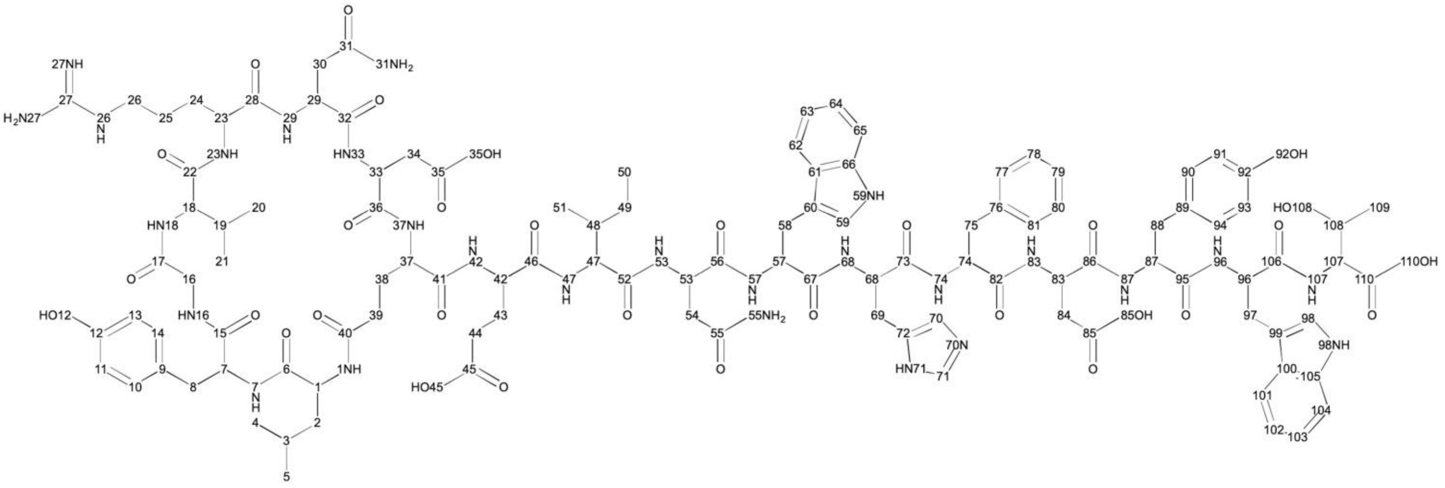

TABLE S1 Oligonucleotides used in this study (sequence 5' to 3', incorporated enzyme restriction sites are underlined)

**Name**	**Sequence**	**Notes**
M13F-24mer	CGCCAGGGTTTTCCCAGTCACGAC	Universal sequencing primers
M13R-22mer	TCACACAGGAAACAGCTATGAC	
JP127_NdeI	CATATGGAACCCCAGATGACTGAG	PCR amplification and cloning in pIJ10257 of the Lp3 BGC
JP128_PacI	TTAATTAACGTGTCGACCGGTGTCAGG	
JP138_NdeI	AACATATGGAACCCTGCGTCCCG	PCR amplification and cloning in pIJ10257 of *leeCEBA* of the Lp2 BGC
JP139_HindIII	AAAAGCTTAGACCCTCATCCGCGCAATG	
JP143_AvrII	AACCTAGGGCGGGGTGACCTGAGGTGGATG	PCR amplification of the Lp2 precursor peptide and transport genes (*leeADF*) for merging with *leeCEBA* as shown in Fig. S5
JP144_NdeI	AACATATGGAGCACGACGAGAAGACG	

Table S2 NMR data table for leepeptin (9.1 mM, DMSO-d_6, 600 MHz, 298 K) For atom numbers see Fig. S11.

Residue	Atom Number	Position	δC/δN, type	δH, mult. [J (Hz)]	Residue	Atom Number	Position	δC/δN, type	δH, mult. [J (Hz)]
Leu1	1	α	52.3, CH	3.99, m	Asn11	53	α	53.2, CH	4.61, m
	2	β	42.0, CH_2	a: 0.61, m		54	β	38.9, CH_2	a: 2.74, m
				b: 0.54, m					b: 2.66, m
	3	γ	23.8, CH	0.60, m		55	γ CO	-	
	4	δ	22.2, CH_3	0.52, m		56	CO	-	
	5	δ*	22.8, CH_3	0.46, m		53NH	NH	-	8.93, br s
	6	CO	-			55NH2	γ-NH2	-	-
	1NH	NH	-	5.94, br s					-
					Trp12	57	α	53.0, CH	4.14, m
Tyr2	7	α	52.3, CH	4.60, m		58	β	28.3, CH_2	a: 3.01, m
	8	β	38.4, CH_2	a: 2.35, m					b: 2.95, m
				b: 1.00, m		60	γ	-	-
	9	γ	128.1, C			59	δ	124.2, CH	7.54, s
	10,14	δ/δ′	130.8, CH	6.71, d (8.0)		66	ε	137.8, C	-

	11,13	ε/ε′	115.0, CH	6.60, d (8.0)		65	ζ	111.9, CH	7.43, d (7.8)
	12	ζ	155.7, C			64	η	122.0, CH	7.17, t (7.2)
	15	CO	-			63	θ	118.8, CH	7.06, t (7.4)
	7NH	NH	-	7.67, d (7.5)		62	ι	120.3, CH	8.28, m
	12OH	ζ-OH	-	-		61	κ	127.6, C	-
						67	CO	-	
Gly3	16	α	42.1, CH_2	a: 4.56, m		57NH	NH	-	7.36, m
				b: 3.53, m		59NH	Indole NH	-	10.94/11.03
	17	CO	-						
					His13	68	α	55.2, CH	4.16, m
	16NH	NH	-	9.1, br s		69	β	25.5, CH_2	a: 2.24, m
									b: 1.05, m
Val4	18	α	59.2, CH	3.95, m		70	γ	CH	-
	19	β	30.3, CH	1.99, m		70N	γN	-	-
	20	γ	19.8, CH_3	0.95, d (6.9)		71	δ	CH	-

	21	γ *	18.8, CH_3	0.93, d (6.9)		71NH	δNH	-	-
	22	CO	-			72	ε	CH	-
	18NH	NH	-	8.03, br s		73	CO	-	
						68NH	NH	-	6.17, br s
Arg5	23	α	58.1, CH	4.53, m					
	24	β	25.6, CH_2	a: 2.03, m	Phe14	74	α	54.0, CH	5.20, m
				b: 1.39, m		75	β	33.2, CH_2	a: 2.45,m
	25	γ	25.5, CH_2	a: 1.18, m					b: 2.00, m
				b: 1.05, m		76	γ	139.0, C	
	26	δ	40.8, CH_2	2.90, m		77, 81	δ/δ′	130.5, CH	7.10, m
	26NH	εNH	-	7.21, m		78, 80	ε/ε′	127.5, CH	7.24, m
	27	CO	-			79	ξ	125.5, CH	7.02, t (7.2)
	27NH2	ηNH2	-	?		82	CO	-	
	27NH	NH	-	?		74NH	NH	-	8.31, br s
	28	CO	-						
	23HN	NH	-	8.29, m	Asp15	83	α	54.0, CH	5.22, m

						84	β	37.9, CH_2	a: 3.08, m
Asn6	29	α	54.0, CH	5.21, m					b: 3.00, m
	30	β	39.0, CH_2	a: 3.37, m		85	γ CO	-	
				b: 3.17, m		86	CO	-	
	31	γ CO	-			83NH	NH	-	8.46, d (6.7)
	32	CO	-						
	29NH	NH	-	7.34, m	Tyr16	87	α	52.2, CH	4.23, m
	31NH2	γ-NH2	-	-		88	β	35.0, CH_2	a: 2.92, m
				-					b: 2.89, m
Asp7	33	α	55.7, CH	4.08, m		89	γ	126.7, C	
	34	β	30.6, CH_2	a: 2.27, m		90, 94	δ/δ′	132.0, CH	7.20, m
				b: 2.11, m		91, 93	ε/ε′	114.8, CH	6.38, d (7.8)
	35	γ CO	-			92	ζ	156.6, C	
	36	CO	-			95	CO	-	
	33NH	NH	-	8.18, br s		87NH	NH	-	8.13, m
						92OH	ζ-OH	-	-

Glu8	37	α	55.0, CH	3.75, m					
	38	β	27.8, CH_2	a: 1.75, m	Trp17	96	α	54.7, CH	4.73, br s
				b: 1.51, m		97	β	29.2, CH_2	a: 3.43, m
	39	γ	33.2, CH_2	1.67, m					b: 3.10, m
	40	δ CO	-			99	γ	110.2, C	-
	41	CO	-			98	δ	124.2, CH	7.37, s
	37NH	NH	-	8.38, br s		105	ε	136.5, C	-
						104	ζ	112.0, CH	7.33, d (7.9)
Glu9	42	α	48.7, CH	4.46, d (8.5)		103	η	121.1, CH	6.82, m
	43	β	24.9, CH_2	a: 2.24, m		102	θ	119.4, CH	6.88, t (7.0)
				b: 2.20, m		101	ι	118.7, CH	7.74, m
	44	γ	37.2, CH_2	3.06, m		100	κ	127.4, C	-
	45	δ CO	-			106	CO	-	
	46	CO	-			96NH	NH	-	9.23, br s
	42NH	NH	-	7.80, d (8.5)		98NH	Indole NH	-	11.03/10.94

Ile10	47	α	63.6, CH	3.40, m	Thr18	107	α	58.1, CH	4.56, m
	48	β	32.0, CH_2	2.67, m		108	β	67.0, CH	4.34, m
	49	γ	24.9, CH_2	a: 1.54, m		109	γ	21.1, CH_3	1.18, d (6.0)
				b: 1.37, m		108OH	βOH	-	-
	50	δ	9.2, CH_3	0.84, t (7.3)		110	CO	-	
	51	ε	15.6, CH_3	0.82, d (6.3)		107NH	NH	-	8.14, m
	52	CO	-						
	47NH	NH	-	8.25, m					

REFERENCES

1. Um S, Kim YJ, Kwon H, Wen H, Kim SH, Kwon HC, Park S, Shin J, Oh DC. 2013. Sungsanpin, a lasso peptide from a deep-sea streptomycete. J Nat Prod 76:873-8799. https://doi. org/doi: 10.1021/np300902g.
2. Elsayed SS, Trusch F, Deng H, Raab A, Prokes I, Busarakam K, Asenjo JA, Andrews BA, van West P, Bull AT, Goodfellow M, Yi Y, Ebel R, Jaspars M, Rateb ME. 2015. Chaxapeptin, a lasso peptide from extremotolerant *Streptomyces leeuwenhoekii* strain C58 from the hyperarid Atacama Desert. J Org Chem 80:10252-10260. https://doi. org/doi: 10.1021/acs.joc.5b01878.
3. Tietz JI, Schwalen CJ, Patel PS, Maxson T, Blair PM, Tai HC, Zakai UI, Mitchell DA. 2017. Nat Chem Biol 13:470-478. https://doi. org/doi: 10.1038/nchembio.2319.
4. Gerlt JA, Bouvier JT, Davidson DB, Imker HJ, Sadkhin B, Slater DR, Whalen KL. 2015. Enzyme Function Initiative-Enzyme Similarity Tool (EFI-EST): A web tool for generating protein sequence similarity networks, Biochimica et Biophysica Acta - Proteins and Proteomics 1854:1019-1037. ISSN 1570-9639, https://dx.doi.org/10.1016/j.bbapap.2015.04.015
5. Shannon P, Markiel A, Ozier O, Baliga NS, Wang JT, Ramage D, Amin N, Schwikowski B, Ideker T. 2003 Cytoscape: a software environment for integrated models of biomolecular interaction networks Genome Research 13:2498-2504. https://doi.org/10.1101/gr.1239303
